# Gliotransmission of D-serine promotes thirst-directed behaviors in *Drosophila*

**DOI:** 10.1016/j.cub.2022.07.038

**Published:** 2022-09-26

**Authors:** Annie Park, Vincent Croset, Nils Otto, Devika Agarwal, Christoph D. Treiber, Eleonora Meschi, David Sims, Scott Waddell

**Affiliations:** 1 Centre for Neural Circuits & Behaviour, University of Oxford, Oxford OX1 3TA, UK; 2 Department of Biosciences, Durham University, Durham DH1 3LE, UK; 3 MRC Computational Genomics Analysis and Training Programme (CGAT), MRC Centre for Computational Biology, MRC Weatherall Institute of Molecular Medicine, John Radcliffe Hospital, Headington, Oxford OX3 9DS, UK

**Keywords:** thirst, single-cell transcriptomics, glia, astrocytes, gliotransmission, D-serine, NMDA receptors, Drosophila, behavior, water procurement

## Abstract

Thirst emerges from a range of cellular changes that ultimately motivate an animal to consume water. Although thirst-responsive neuronal signals have been reported, the full complement of brain responses is unclear. Here, we identify molecular and cellular adaptations in the brain using single-cell sequencing of water-deprived *Drosophila*. Water deficiency primarily altered the glial transcriptome. Screening the regulated genes revealed astrocytic expression of the *astray*-encoded phosphoserine phosphatase to bi-directionally regulate water consumption. Astray synthesizes the gliotransmitter D-serine, and vesicular release from astrocytes is required for drinking. Moreover, dietary D-serine rescues *aay*-dependent drinking deficits while facilitating water consumption and expression of water-seeking memory. D-serine action requires binding to neuronal NMDA-type glutamate receptors. Fly astrocytes contribute processes to tripartite synapses, and the proportion of astrocytes that are themselves activated by glutamate increases with water deprivation. We propose that thirst elevates astrocytic D-serine release, which awakens quiescent glutamatergic circuits to enhance water procurement.

## Highlights

• Water deprivation alters the levels of many glial-expressed genes in *Drosophila*
• Levels of *astray*-encoded phosphoserine phosphatase bi-directionally alter drinking
• Astrocytic D-serine regulates water procurement via NMDA-type glutamate receptors
• Astrocytes frequently contribute processes to tripartite glutamatergic synapses

## Graphical abstract

**Figure:**
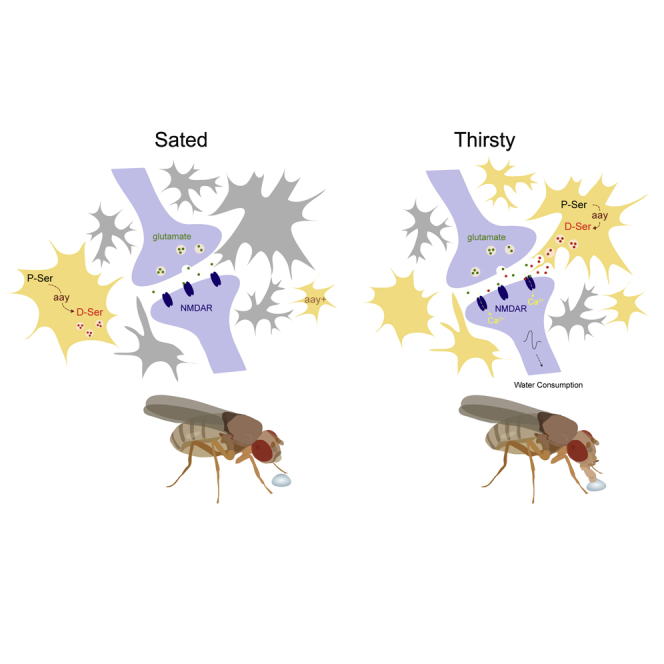

## Teaser

Park et al. show that water deprivation primarily alters the glial transcriptome in *Drosophila*. Screening the regulated genes identified astrocytic expression of the *astray*-encoded phosphoserine phosphatase and astrocyte-released D-serine to be key elements of the brain’s response toward facilitating thirst-directed behaviors.

## Introduction

The sensation of thirst is predominantly a manifestation of our body’s response to being water deprived. Water deficit reduces the volume and increases the osmolarity of an animal’s blood. These changes induce multiple adaptations throughout the body, such as elevated activity of osmosensory neurons in the brain, alteration of blood pressure and heart rate, and increased retention of water.

Osmosensory neurons in the subfornical organ (SFO) and organum vasculosum (OV) of the lamina terminalis (LT) of the mammalian forebrain respond directly and indirectly to changes in plasma osmolality and blood volume/pressure, via hormones such as Angiotensin II.^1^, ^2^, ^3^ These neurons then directly or indirectly release compensatory neuropeptide/hormone signals like Vasopressin/anti-diuretic hormone which promotes bodily responses to reduce water loss and restore blood pressure, and behavioral responses to restore fluid balance.^4^, ^5^, ^6^, ^7^ Artificial engagement of LT neurons can induce water consummatory behaviors.^8^, ^9^, ^10^, ^11^, ^12^, ^13^, ^14^, ^15^, ^16^, ^17^ However, the full range of nervous system mechanisms that accompany the water-deprived state, and how they modulate behavioral regimes and actions to satisfy thirst, is currently unknown.

In *Drosophila*, ion transport peptide (ITP) is the likely anti-diuretic functional analog of the mammalian vasopressin and renin-angiotensin systems.^18^ ITP is produced by neurosecretory neurons in the brain and abdominal ganglion; its expression increases with water deprivation. ITP increases water consumption, reduces water excretion in the malpighian tubules, and increases hindgut reabsorption. ITP induction also represses feeding. Although circuits regulated by ITP remain to be identified, other thirst-regulated circuits required for water seeking and consumption/homeostasis have been reported. Interoceptive sensory neurons (ISNs) in the subesophageal zone (SEZ) directly sense high osmolality (via the cation channel Nanchung) and their inhibition promotes drinking.^19^ Interestingly, ISN activation (via adipokinetic hormine [AKH]) suppresses drinking and instead promotes feeding. Two other classes of SEZ neurons, the Janu neurons, regulate water seeking behavior up a humidity gradient, but not total water consumption.^20^ The Janu neurons are either GABAergic or Allatostatin (AstA)-releasing. Their joint activation is rewarding, and the AstA group simultaneously inhibits feeding while promoting water seeking. Finally, different types of mushroom body innervating dopaminergic (DA) neurons have been implicated in water seeking, water reward learning,^21^ and thirst-dependent control of the expression of water-seeking memories.^22^ Some of these DA neurons are regulated by the leucokinin neuropeptide which is released from neurons that are activated by elevated osmolality.^22^ More complex interaction between neuromodulators allows DA neurons to selectively promote state appropriate expression of thirst- or hunger-dependent memories.

Brain responses to water deprivation need not be exclusively neuronal. A blood-brain barrier (BBB) shields most neurons from the circulatory environment. Notably, the osmosensory neurons of the mammalian SFO and OV have processes outside the BBB where they can directly sample circulatory status.^23^ The mammalian BBB is formed by microvascular endothelial cells, pericytes, and astrocytes. Since astrocytes also innervate the neuropil, where their processes contribute to tripartite synapses (TPSs), they are well positioned to sense and/or transmit metabolites and signals representing nutrient status to neurons.^24^, ^25^, ^26^, ^27^, ^28^, ^29^, ^30^ In *Drosophila*, perineurial and subperineurial glia form the BBB, whereas astrocytes tile the entire brain and permeate the neuropil.^26^^,^^31^ Astrocytes in the fly therefore also have potential to influence neuronal activity in response to nutritional state.^32^ However, evidence for such a role of fly astrocytes is currently lacking.

The *Drosophila* brain provides an unparalleled opportunity to investigate neural mechanisms of thirst with cellular and molecular resolution. Recent advances in single-cell transcriptomics have generated transcriptional profiles from most of the abundant cell types in the fly brain.^33^, ^34^, ^35^, ^36^, ^37^ Here, we used single-cell transcriptomics to identify brain-wide and cell-type restricted changes in gene expression triggered by water deprivation. Most changes occurred within glia, rather than neurons. Functional analyses of thirst responsive genes identified astrocyte expression of the *astray* (*aay*)-encoded phosphoserine phosphatase and gliotransmission of its product D-serine to be required for regulated water consumption. We show that astrocytes contribute processes to tripartite glutamatergic synapses in the fly brain and that D-serine promotes water procurement via fly NMDA-type glutamate receptors. These findings provide a new molecular and cellular framework to understand how thirst alters brain physiology and behavior.

## Results

### Single-cell transcriptomics in thirsty *Drosophila*

**Figure 1:**
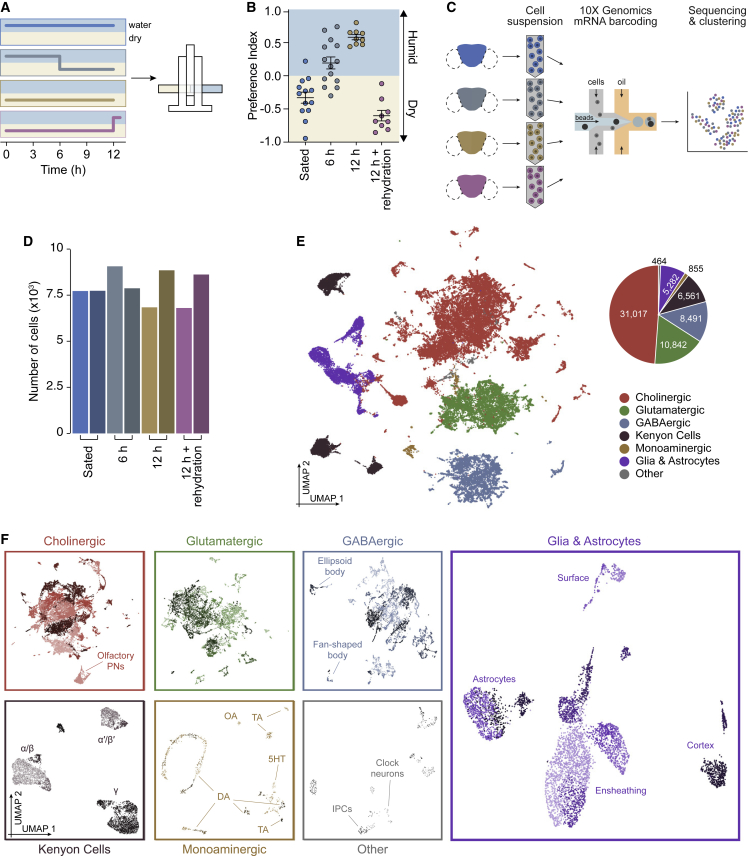
Single-cell transcriptomics of thirsty *Drosophila* (A) Schematic protocols for humidity preference assay. Flies were kept in vials with or without water for the indicated times, then given a T-maze choice between a humid and a dry chamber. (B) Increasing dehydration converts humidity avoidance behavior into attraction. Attraction returns to avoidance with thirst quenching. (C) Schematic of the process of single-cell transcriptomics analyses comparing flies from the four conditions in (B). Two independent samples were processed for each condition. (D) Total number of cells obtained from each sample, after filtration of low-quality barcodes and doublets. (E) Left: UMAP plot from first clustering step, and identification of seven main cell classes. Right: pie chart showing the number of cells obtained from each of these classes. (F) UMAP plots showing sub-clustering of each of the seven cell classes shown in (E). Known cell types within each class are labeled. Kenyon cell labels represent known subtypes that innervate corresponding mushroom body lobes. Withing the monoaminergic cells labels are OA, octopaminergic; TA, tyraminergic; 5HT, serotonergic; DA, dopaminergic. Within other, IPCs, insulin-producing cells. See also Figure S1.

*Drosophila* prefer dry environments when water sated but seek humidity when water deprived, behaviors that serve fluid homeostasis.^21^^,^^38^^,^^39^ This behavioral switch is readily quantified by giving flies the choice in a T-maze between humid and dry chambers (Figure 1A). Although water-sated flies preferred the dry, those dehydrated for 6 or 12 h showed an increasing preference for humidity (Figure 1B). Flies water deprived for 12 h then permitted to drink reverted to preference for the dry chamber, demonstrating water attraction is rapidly reversed upon drinking (Figures 1A and 1B).

To investigate cellular correlates of thirst, we used the 10X Genomics Chromium system to generate single-cell transcriptomic atlases of brains extracted from water-sated, 6 and 12 h dehydrated, and rehydrated flies (Figure 1C). After filtering out low-quality barcodes and putative cell doublets (Figure S1A), we retrieved a total of 63,512 cells. Importantly, each dehydration condition and experimental sample contributed a similar cell number to the total collection (Figure 1D). We used SCTransform and the canonical correlation anchor-based method in Seurat v3^40,41^ to normalize and integrate data from each sample to help identify shared cell populations. An initial unsupervised clustering step was performed to partition cells into seven main classes, which we annotated based on the expression of known marker genes (Figure S1B). These classes include cholinergic, glutamatergic, GABAergic, mushroom body intrinsic Kenyon cells, monoaminergic neurons, glia, and “other” cells not in these previous categories (Figure 1E).^33^^,^^40^ Cells in each class were next independently subdivided into 184 clusters, each containing between 14 and 5,083 cells (Figure 1F). Monoaminergic clusters were smaller than others (median cells/cluster: 32), reflecting their discrete transmitters (DA, octopaminergic [OA], tyraminergic [TA], serotonergic [5HT]), functional specialization among neurons of each type, and relative scarcity in the brain. Conversely, Kenyon cell and other cholinergic neuron clusters were largest (median cells/cluster: 354.5 and 293.5, respectively; Figure S1C). Again, for each of these 184 clusters, we identified specific marker genes (Data S1; Figure S1F), with which we annotated known cell types (Figure 1F; see STAR Methods for detail). We noted that each experimental sample was evenly represented across cell clusters (Figures S1D and S1E), indicating that dehydration does not change the cellular composition of the adult fly brain, in contrast to in developing larvae where starvation reduced the number of undifferentiated *headcase* expressing neurons.^41^

### The transcriptional signature of thirst in the fly brain

**Figure 2:**
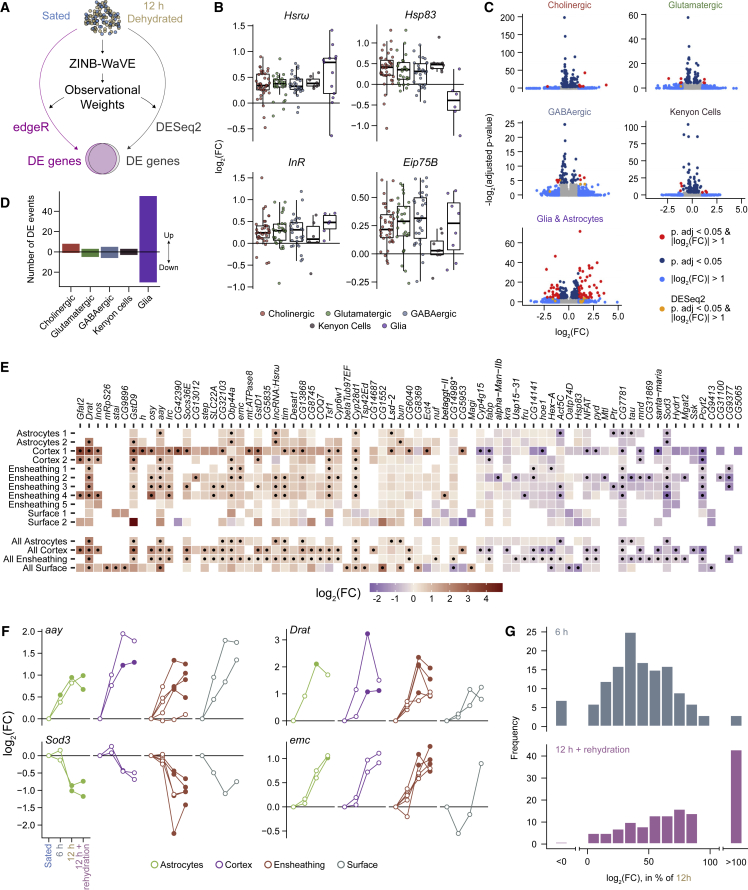
The transcriptional signature of thirst in the fly brain (A) Schematic of differential expression analysis. Observational weights calculated with ZINB-WaVE were used in edgeR and DESeq2 to correct for zero-inflation. (B) Boxplots showing differential expression of the four genes most broadly regulated in each cluster after 12 h dehydration, grouped by main cell class. (C) Volcano plots representing statistical significance against fold change for all genes tested in cholinergic neurons, glutamatergic neurons, GABAergic neurons, Kenyon cells, and glia, calculated with edgeR. Each plot represents pooled data from all clusters in each cell class. Genes with adjusted p value < 0.05 and |log_2_(FC)| > 1 in DESeq2 but not edgeR are labeled orange. (D) Number of differential expression events identified in the five cell classes shown in (C). Values above the bar represent genes upregulated in thirsty flies and below the bar represents downregulated genes. (E) Heatmap showing fold change for each of the most regulated genes in glia, for each cluster (top) and for clusters grouped by glia type (bottom), calculated with edgeR. Black dots: adjusted p value < 0.05. Empty tiles: transcript levels below detection threshold. ^∗^: *CG14989* is the only gene significantly upregulated and downregulated in different clusters. (F) Changes in expression for four differentially expressed genes across glia clusters, and through all four hydration conditions. In most cases, expression gradually increases or decreases during dehydration. After rehydration, mRNA levels tended to remain similar to levels measured in dehydrated flies. Full circles indicate adjusted p value < 0.05, and empty circles indicate adjusted p value ≥ 0.05. (G) Comparisons of log_2_(FC) values (versus sated controls) after 6 h dehydration (top) or after rehydration (bottom), as a percentage of log_2_(FC) values after 12 h dehydration. Percentages were calculated for each of the significant gene-cluster pairs shown in (E) (black dots) and represented in histograms in 10% increments. Changes in expression are lower (<100%) after 6 h than after 12 h dehydration (top) for most genes. However, expression of many genes remains high (>100%) after thirsty flies are allowed to drink (bottom). See also Figure S2.

A characteristic of single-cell RNA sequencing (scRNA-seq) data is that expression of certain genes is stochastically undetectable in a fraction of the cells in which they are normally expressed. This artefact called “dropout” can result from low mRNA levels in single cells and inefficient mRNA capture during library preparation, and it produces a larger than expected number of zero read counts.^42^^,^^43^ Therefore, classic differential expression methods designed for negative binomial bulk transcriptome data often underperform when analyzing weakly expressed genes in scRNA-seq data.^44^^,^^45^ To correct for potential bias caused by zero read count inflation, we downweighed excess zeros using ZINB-WaVE,^46^^,^^47^ which enables a more accurate estimation of data dispersion and thereby improves detection of differentially expressed genes. Using ZINB-WaVE with both edgeR and DESeq2, two tools for quantifying differential expression,^48^^,^^49^ we calculated gene expression differences between water sated and 12 h dehydrated conditions across cell clusters (Figure 2A).

We expected that general osmotic stress of dehydration might trigger a brain-wide transcriptional response. We identified four genes whose expression increased across most clusters, with an average log_2_(FC) across clusters above 0.25 (Figure 2B). Two of these, the chaperone encoding *Hsp83* and the long non-coding RNA *Hsrw*, have been implicated in the cellular stress response and shown together to regulate transcription.^50^, ^51^, ^52^ Global upregulation of the insulin receptor (*InR*) may reflect the role of insulin signaling in balancing thirst and hunger,^19^^,^^53^ whereas the ecdysone-response gene *Eip75B* buffers circadian rhythms in stressful conditions.^54^

Cell-types exhibiting state-dependent changes in gene expression potentially play a role in the fly’s response to thirst, and the regulated genes may represent intra or intercellular mechanisms mediating physiological and behavioral responses. 84 genes showed strong differential expression (|log_2_(FC)| > 1, adjusted p value < 0.05) in at least one cell cluster, with either edgeR or DESeq2, which identified slightly different sets of genes (Figure S2A). Although we detected fewer genes in glia than in other cell clusters (Figure S2D), most differential expression events (85/119) occurred in glia, with the rest in cholinergic, glutamatergic, GABAergic, and Kenyon cell classes (Figures 2C and 2D). No individual cluster within monoaminergic and “other” classes contained enough cells to enable differential analysis with sufficient statistical power. No differences were apparent when clusters from these two classes were analyzed as a group. Within the glial clusters, 6 genes passed our differential expression criteria in astrocytes, 26 in ensheathing glia, and 33 in cortex glia. No differences were found in surface glia. However, pooling glial clusters by type provided enough statistical power to reveal 15 genes in surface glia and 2 additional genes in cortex glia (Figure 2E). With the exception of *CG14989*, which was upregulated in ensheathing glia and downregulated in surface glia, all other differentially expressed genes changed in a similar direction in multiple glial types. Gene Ontology analysis indicated that differentially expressed genes contribute to pathways related to metabolism, response to stress, or behavior (Figure S2E). In summary, the highest magnitude thirst-dependent changes in gene expression occur in glial cell-types, whereas neuronal transcriptomes remain comparatively stable.

We next tested whether expression of thirst-responsive genes changed with dehydration time. For several, including *aay*, *Death resistor Adh domain containing target* (*Drat*) and *extra macrochaetae* (*emc*), expression steadily increased as dehydration progressed (Figure 2F), tracking the gradual increase in behavioral preference for humidity (Figure 1B). Indeed, a majority of log_2_(FC) values for 6 h were between 30% and 70% of those at 12 h (Figure 2G). In contrast, *superoxide dismutase 3* (*Sod3*) expression remained stable until 6 h but decreased by 12 h (Figure 2F, bottom left) consistent with its suppression, perhaps participating in the metabolic or stress response to severe dehydration. Quenching thirst tended to return some transcript levels toward their sated baseline, although with different magnitude for different genes and clusters. However, transcript levels of many genes continued to increase after rehydration (Figure 2F, bottom right, Figure 2G), suggesting prolonged action beyond the rehydration period. Together, these results illustrate a diversity of water deprivation-induced gene regulation patterns.

### Astrocytic *aay* bi-directionally regulates water consumption

**Figure 3:**
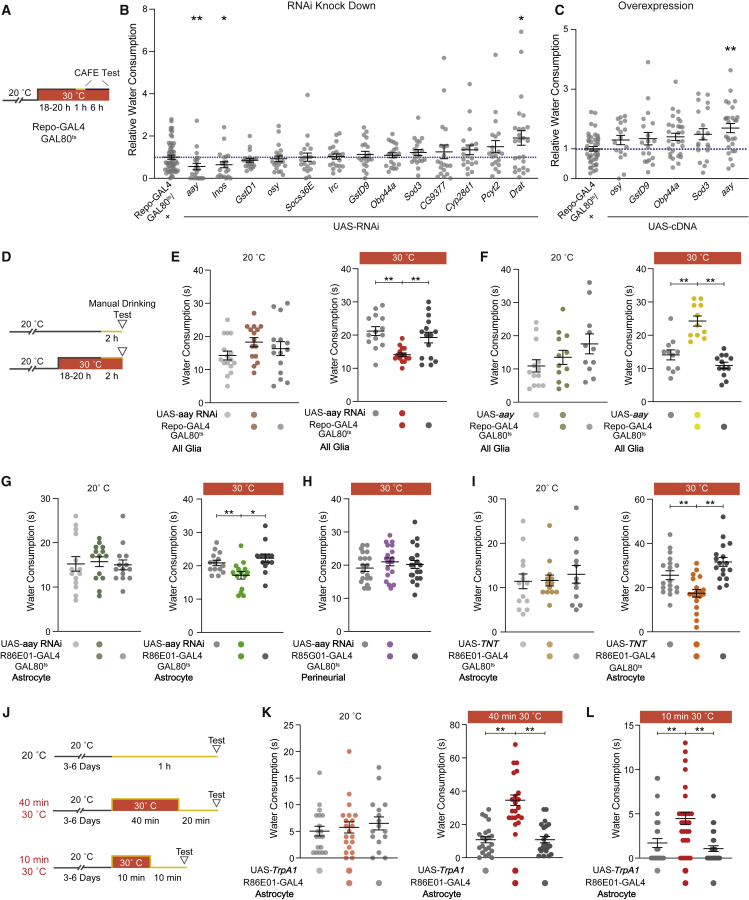
Astrocytic *aay* is a novel regulator of water consumption (A) Schematic for temperature control of GAL80^ts^/GAL4 driven expression of UAS-RNAi or UAS-cDNA transgenes with CAFE test. Orange section of line indicates period of water restriction. (B) Water consumption in the CAFE assay of flies with RNAi knock down of candidate genes. Dotted blue line indicates normalization to control Repo-GAL4 flies, equal to 1. ^∗^p < 0.05, ^∗∗^p < 0.01 two-tailed Mann-Whitney test n_RNAi_ = 20–25, n_Repo-GAL4_ = 50. (C) Water consumption in CAFE for UAS overexpression of targets. ^∗∗^p < 0.01 two-tailed Mann-Whitney test n_RNAi_ = 17–29, n_Repo-GAL4_ = 43. (D) Schematic for temperature control of GAL80^ts^/GAL4-driven UAS-RNAi or UAS-cDNA transgenes with manual water feeding assay. Orange section of line indicates period of water restriction. (E) RNAi knockdown of *aay* reduces water consumption (n = 14–16). (F) Overexpression of *aay* increases water consumption (n = 11–13). (G) Astrocyte-specific RNAi knockdown of *aay* reduces water consumption (n = 13 and 14). (H) RNAi knockdown of *aay* in perineurial glia does not alter water consumption (n = 18–20). (I) Preventing vesicular transmission with tetanus-toxin (TNT) expression in astrocytes reduces water consumption (n = 12–19). (J) Temperature regimen for TrpA1 activation of astrocytes in (K) and (L). Orange section of line indicates period of water restriction. (K) Astrocyte activation for 40 min increases water consumption (n = 16–22). ^∗∗^p < 0.01, Kruskal-Wallis ANOVA with Dunn’s multiple comparisons test. (L) Astrocyte activation for 10 min increases water consumption (n = 28–30). ^∗∗^p < 0.01, Kruskal-Wallis ANOVA with Dunn’s multiple comparisons test. Normality was assessed using Shapiro-Wilk test. ^∗^p < 0.05, ^∗∗^p < 0.01, ^∗∗∗^p < 0.001, ^∗∗∗∗^p < 0.0001 Ordinary one-way ANOVA with Dunnett’s multiple comparisons test, unless otherwise stated. Individual data points are single flies. Data are mean ± standard error of the mean (SEM). See also Figure S3.

We tested whether glial genes identified by scRNA-seq regulated water consumption in the capillary feeding (CAFE) assay^55^ (Figures 3A and 3B). We targeted temporally restricted RNAi expression to adult glia using Repo-GAL4 combined with a ubiquitously expressed temperature-sensitive GAL80^56^ (Figure 3C). The strongest change in water consumption occured with *aay*. Adult-restricted RNAi knockdown of *aay* reduced water consumption, whereas overexpression increased it (Figures 3B, 3C, S3D, and S3E). Notably, *aay* RNAi appears to specifically regulate water consumption, as feeding was unaffected (Figure S3F).

We noticed significant death in the CAFE assay when manipulating *aay* (Figures S3G, S4D, and S4E), perhaps from sensitivity to stress of dehydration, or idiosyncrasies of the assay.^57^ To circumvent this issue, we employed a manual water consumption assay that is less challenging for the animals.^19^ These experiments revealed similar results to CAFE when knocking down or overexpressing *aay* in glia (Figures 3D–3F). We next tested whether *aay*-induced changes in water consumption could be assigned to a glial cell type.^58^ Expressing *aay* RNAi in astrocytes, but not in perineurial glia, reduced water consumption indicating the specific importance of astrocytic *aay* in controlling drinking (Figures 3G and 3H). Although baseline drinking of control flies varied between experiments, relative differences between genotypes and treatments remained consistent.

The *aay* gene encodes a phosphoserine phosphatase which converts phosphoserine into D- and L-serine.^59^, ^60^, ^61^ In mammals, the gliotransmitter D-serine is packaged into astrocytic vesicles and released in a SNARE and calcium-dependent manner.^62^, ^63^, ^64^, ^65^, ^66^ *Drosophila* astrocytes express vesicular machinery including synaptobrevin (*Syb*) and Syntaxin 1A (*Syx1A*) (Figures S3M and S3N). We tested whether vesicular release from astrocytes is necessary for water consumption by expressing a tetanus-toxin (TetX) transgene using an astrocyte-specific GAL4. Tetx abolishes evoked release by cleaving the vesicle-associated membrane protein synaptobrevin. Temporally restricted TetX expression in astrocytes reduced water consumption (Figure 3I).^58^

We also tested whether evoking Ca^2+^ entry into astrocytes promoted water consumption by expressing a transgene encoding the Ca^2+^-permeable temperature-sensitive TrpA1 channel in astrocytes (*R86E01*>*TrpA1*). We first tested flies with a 2 h “fictive dehydration” step at the TrpA1 activation temperature of 30°C; however, this manipulation reversibly paralyzed *R86E01*>*TrpA1* flies, as previously described.^67^ We therefore shortened the 30°C stimulation to 40 min and allowed flies to recover at 20°C for 20 min (Figure 3J). This procedure substantially increased water consumption compared to controls carrying the *R86E01-GAL4* or UAS-*TrpA1* transgenes or those left at 20°C for the whole procedure (Figure 3K). Since vesicular release occurs promptly following astrocytic activation,^62^ we also further restricted the heat incubation to 10 min. This brief astrocyte activation was sufficient to increase water consumption (Figures 3L and S3L). Together, these data indicate that *aay* expression and vesicular release from activated astrocytes regulate water consumption.

### D-serine facilitates water consumption via NMDA receptors

**Figure 4:**
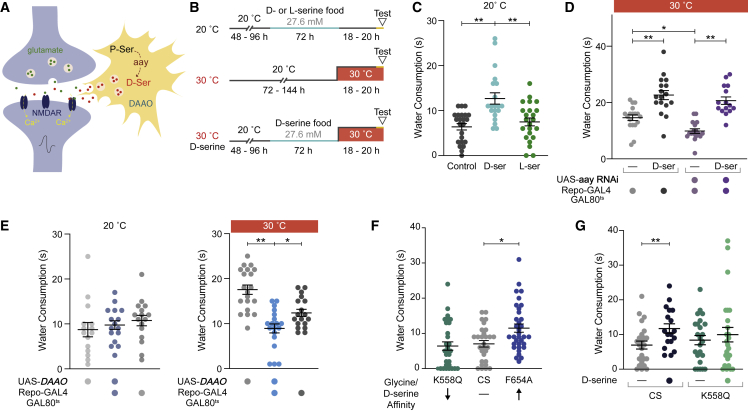
D-serine modulates water consumption via NMDARs (A) Model of tripartite glutamatergic synapse showing *aay*-dependent synthesis of D-serine in astrocytes. (B) Protocol for D-serine feeding and RNAi induction with water consumption experiments. (C–G) Light blue section of lines indicate time on D- or L-serine food. Orange section of lines indicate period of water restriction. (C) D- but not L-serine feeding increases water consumption (n = 20–23). ^∗∗^p < 0.01, Kruskal-Wallis ANOVA with Dunn’s multiple comparisons test. (D) D-serine feeding rescues the water consumption defect in flies with *aay* knockdown (n = 15–18). ^∗^p < 0.05, ^∗∗^p < 0.01, ordinary one-way ANOVA with Dunnett’s multiple comparisons test. (E) Glial overexpression of D-amino acid oxidase (*DAAO*) reduces water consumption (n = 16–20). ^∗^p < 0.05, ^∗∗^p < 0.01, ordinary one-way ANOVA with Dunnett’s multiple comparisons test. (F) Flies harboring the F654A single site mutation in NMDAR1 exhibit increased water consumption. This mutation increases affinity for glycine/D-serine (n = 29–34). ^∗^p < 0.05, Kruskal-Wallis ANOVA with Dunn’s multiple comparisons test. (G) Flies harboring the K558Q amino acid substitution in NMDAR1 are insensitive to the D-serine induced increase in water consumption (n = 18–25). ^∗∗^p < 0.01, two-tailed Mann-Whitney test. Individual data points are single flies. Data are mean ± SEM. See also Figure S4.

To further assess the role of D-serine as a gliotransmitter in water consumption, we supplemented the flies’ diet with D-serine and measured water intake (Figures 4A, 4B, and S4A). Feeding flies with D- but not L-serine increased water consumption (Figure 4C). D-serine is an enantiomer-specific co-agonist for NMDA-type glutamate receptors (NMDARs).^68^^,^^69^ We therefore tested if *aay*-dependent drinking defects could be rescued with dietary D-serine. Glial expression of *aay* RNAi was induced in flies previously fed with medium supplemented with D-serine (Figure 4B). D-serine feeding was sufficient to restore the *aay*-induced drinking deficit to a normal level (Figure 4D). Control experiments showed that flies did not prefer D-serine containing food and that D-serine did not alter feeding (Figures S4B and S4C).

D-serine is degraded by D-amino acid oxidase (DAAO), which is encoded by the *CG12338* and *CG11236* genes in *Drosophila*.^70^^,^^71^ Since *CG11236* expression is barely detectable in the brain, we focused on *CG12338*.^70^ Overexpressing a *CG12338* DAAO transgene in glia suppressed water consumption (Figure 4E). Together, the *aay* manipulation, dietary supplementation, and DAAO data suggest that D-serine can bi-directionally regulate water consumption.

NMDARs are voltage and ligand-gated ion channels. Efficient channel activation requires binding of L-glutamate to the NR2 subunit and a co-agonist glycine or D-serine, which occupy the same site on the NR1 subunit.^72^ Prior neuronal depolarization is also required to remove a Mg^2+^ block, allowing ligand-gated channel conductance to occur. We therefore tested whether D-serine facilitated water consumption via NMDAR activation. We used flies harboring single-site mutations (K558Q and F654A) in the *NMDAR1* gene which encodes the fly NR1 subunit. These amino acid substitutions are equivalent to K544Q and F639A in mammalian NR1.^73^, ^74^, ^75^ Glycine affinity is increased in receptors bearing F639A/F654A and decreased in those with K544Q/K558Q, whereas glutamate affinity is unaltered.^76^^,^^77^ Measuring water consumption revealed that flies harboring the F654A substitution in NR1 drank more than controls (Figure 4F). In addition, K558Q flies exhibited normal water consumption but were unable to acquire the D-serine induced increase in drinking (Figure 4G). Therefore, NMDAR co-agonist affinity directly impacts water consumption.

### D-serine is a co-agonist of the *Drosophila* NMDA receptor

**Figure 5:**
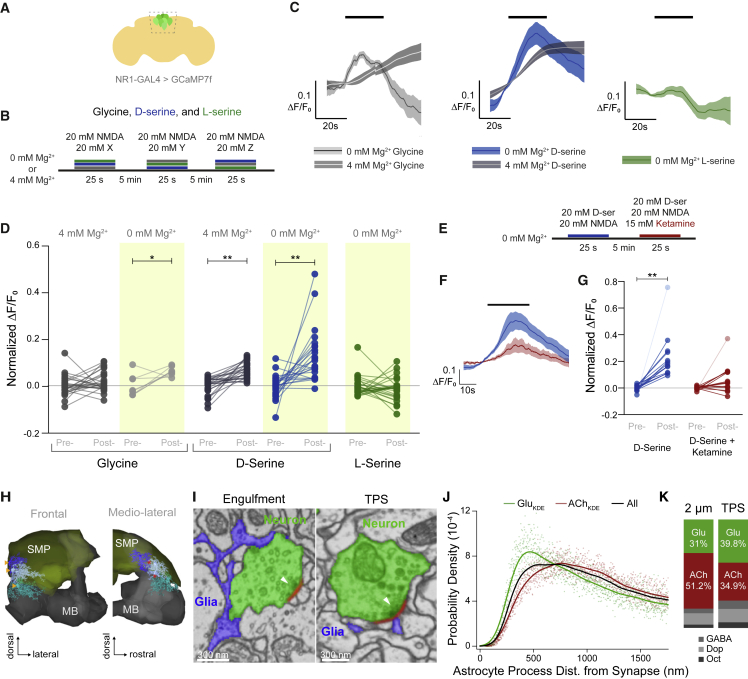
Astrocytes form tripartite synapses and D-serine is a co-agonist for NMDARs (A) Illustration of imaging window for recording of Ca^2+^ responses in NMDAR1 expressing PI neurons. (B) Protocol of drug application for (C) and (D). Order of application was randomized for each fly. (C) Average traces for glycine, D-serine, and L-serine application in 0 or 4 mM Mg^2+^ (n_gly_ = 8, 25; n_D-ser_ = 25, 26; n_L-ser_ = 28, respectively). Line is a moving average and shaded band is SEM. (D) D-serine, but not L-serine, activates NR1+ PI neurons (from left to right, n = 25, 25, 8, 8, 25, 25, 23, 23, 28, and 28). ^∗^p < 0.05, ^∗∗^p < 0.001, Kruskal-Wallis ANOVA with Dunn’s multiple comparisons test. (E) Protocol for ketamine application with NMDA, TTX, and D-serine. (F) Averaged traces for D-serine and NMDA (blue) and D-serine, NMDA, and ketamine (red) (n = 15). (G) Ketamine inhibits D-serine induced activation of NR1+ PI neurons. ^∗∗^p < 0.01, Kruskal-Wallis ANOVA with Dunn’s multiple comparisons test. Transparent dots indicate outliers that are >2 SD from the mean. When outliers are excluded from the analysis, the relationship still holds (see Figure S5F). (H) Astrocytes tile the SMP. 3D representation of three astrocytes (shades of blue) reconstructed from EM data shown with the mushroom body neuropil (gray) and the SMP neuropil (yellow), shown in frontal and lateral views. Cell bodies are located outside the neuropil (yellow arrow heads), and processes in the neuropil have little overlap (^∗^). (I) Astrocytes engulf synaptic boutons and contribute processes to tripartite synapses (TPSs). Grayscale EM data with labeled glutamatergic boutons (green) and glial processes (blue). Presynaptic densities (arrow) and synaptic cleft (red) are indicated. Left: example where glial processes contact a large proportion of a bouton’s membrane but not the synapse. Right: example of a glial process directly adjacent to the synaptic cleft and opposite the presynaptic density (white arrow head). (J) Astrocytic processes are significantly closer to glutamatergic than cholinergic synapses in the SMP. Kernel density estimates (lines) of the probability distributions (points) of distances of Glu or Ach synapses or a random draw from both sets to 3 SMP based astrocytes. Only synapses in direct vicinity (2 μm radius around processes <600 nm thick) are considered. Statistical analyses shown in Figure S5. (K) Glutamatergic synapses are overrepresented in tripartite synapses versus their representation in synapses in the general 2 μm vicinity of astrocytic processes. Ratios of synapses by predicted neurotransmitter usage are shown. See also Figure S5 and Video S1 for anatomy of astrocyte processes engulfing a presynapse and contributing to TPS.

A prior study demonstrated that D- or L-Serine feeding regulated sleep in flies and that the effect depended on NMDARs.^70^ However, it is currently assumed that D-serine is a co-agonist of *Drosophila* NMDARs, as in mammals. Since NMDAR activation elicits increases in intracellular Ca^2+^, we expressed GCaMP7f broadly in NR1 positive (NR1+) neurons and performed *in vivo* calcium imaging in the brain of head fixed flies while bath-applying glycine, D-serine, or L-serine (Figures 5A and 5B). We recorded from NR1+ neurons (*nmdar1-KIGAL4>GCaMP7f*) in the pars intercerebralis (PI) because they are easily accessed and identified and therefore permitted reproducible measurements between flies (Figure 5A).^70^ In addition, responses were measured in Mg^2+^ free (0 mM Mg^2+^) and physiologically relevant concentrations of Mg^2+^ (4 mM Mg^2+^) (Figure 5B) to assess the importance of removing the Mg^2+^ occlusion from the channel pore.^78^^,^^79^ Bath application of NMDA with D-, but not L-, serine activated PI NR1+ neurons (Figures 5C and 5D). NMDA and glycine also produced excitation but only in Mg^2+^ free conditions (Figure 5D). D-serine and glycine in 4 mM Mg^2+^ promoted slower activation, likely due to the voltage-dependent Mg^2+^ block preventing fast activation. To validate that D-serine activation was occurring via NMDARs, we applied the non-competitive NMDAR antagonist ketamine with NMDA and D-serine (Figure 5E).^80^ Ketamine abrogated PI neuron responses to D-serine/NMDA (Figures 5F, 5G, and S5H) confirming that fly NMDARs are activated by D-serine.

Video S1. Fine astrocytic processes often engulf glutamatergic synaptic boutons or contribute to tripartite synapses, related to Figures 5I and S5L 3D representations of an astrocyte (blue) in the SMP and glutamatergic neurons (green) with boutons containing annotated presynapses (red), which consist of synaptic vesicles (red spheres) and pre-synaptic T-bar densities (black). 00:00 to 00:55, When glial cells engulf axons, multiple processes of different sizes contact a substantial area of a presynaptic bouton’s membrane but do not extend into the synapse. 00:56-1:40, Fine astrocyte processes either pass through the synaptic cleft, or terminate within it, to form a tripartite synapse. In both cases the astrocyte processes contact the synaptic cleft alongside the postsynaptic neurons. Many closely spaced boutons of the same presynaptic neuron can be contacted by the same astrocyte, which therefore contributes processes to multiple tripartite synapses. Anatomical features are further highlighted in the video. Cellular meshes were retrieved from FlyWire and animations produced with blender.

### Astrocytes contribute to tripartite synapses in the *Drosophila* brain

Processes from mammalian astrocytes infiltrate the synaptic cleft, forming TPSs.^81^ This allows glia to participate in neurotransmission through transmitter uptake and recycling and by release of gliotransmitters.^82^ *Drosophila* adult neuromuscular synapses have a tripartite structure,^83^ but the detailed anatomy of adult central nervous system (CNS) astrocytes is unclear. To address this, we analyzed astrocytes in the superior medial protocerebrum (SMP) neuropil within the FlyWire project (flywire.ai)^84^ which utilizes the full adult female brain (FAFB) transmission electron microscope dataset.^85^ We fully reconstructed 11 astrocytes and four were extensively reviewed to reconstruct their finest processes. These astrocytes tile the neuropil and closely associate with trachea, as previously reported in the larval brain and ventral nerve cord (VNC)^31^^,^^86^^,^^87^ (Figures 5H, S5J, and S5K). We identified astrocytic processes that engulfed synaptic boutons and that contacted synaptic clefts like processes from postsynaptic neurons—the latter providing evidence of canonical TPSs in the adult CNS (Figures 5I and S5L). Astrocytes also often contact the postsynaptic neuron within 500 nm of the synaptic cleft.

To determine whether astrocytes associate with a particular class of synapse, we used the prior computational predictions of neurotransmitter identity for neurons in the FAFB volume.^88^ We composed a vicinity profile by measuring distances of synapses from the fine processes of the 3 extensively reviewed SMP astrocytes and plotted their distance distributions categorized by presynaptic neurotransmitter. This analysis revealed astrocytic processes to be significantly closer to glutamatergic (962 ± 2 nm SD) and GABAergic (971 ± 3 nm) than cholinergic synapses (1,061 ± 1 nm) (Figures 5J, 5K, S5M, and S5N). Astrocytes are therefore ideally positioned to modulate glutamatergic synapses in the adult brain.

### Astrocytes show heterogeneous responses to neurotransmitters

**Figure 6:**
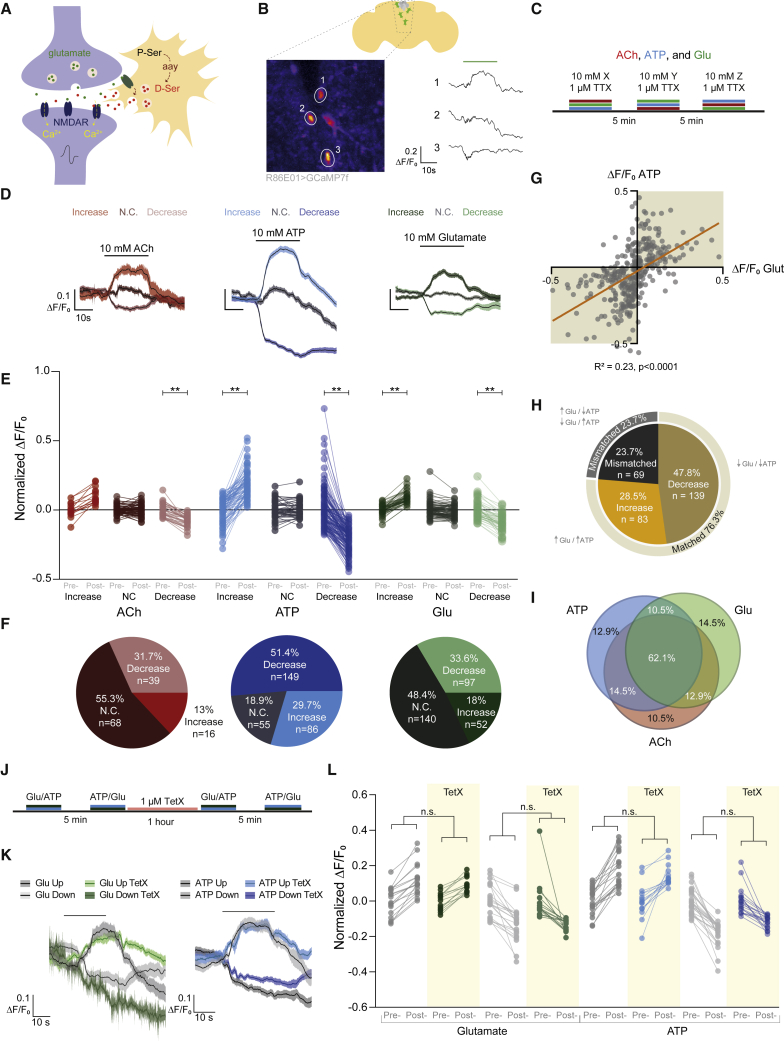
Astrocytes are differentially responsive to neurotransmitters (A) Illustration of possible glutamate-evoked D-serine release from astrocytes. (B) Astrocytes adjacent to the pars intercerebralis show differential responses to glutamate application (green bar). (C) Protocol for drug application with randomized order for acetylcholine (ACh), ATP, and glutamate (Glu). (D) Average traces for ACh, ATP, and glutamate partitioned by the type of response (activated, no change, or inhibited). Responses were determined as follows: activated if μ_post-drug_ > σ_pre-drug_ + μ_pre-drug_, no change if μ_post-drug_ fell within σ_pre-drug_ + μ_pre-drug_ and inhibited if μ_post-drug_ < σ_pre-drug_ − μ_pre-drug_. Line is smoothed average and shaded band is SEM. (E) Neurotransmitters induce both excitatory and inhibitory responses in astrocytes. Paired datapoints represent responses from a single astrocyte, and group is assembled from multiple flies (n_Ach_ = 16, 68, 39; n_ATP_ = 86, 55, 149; n_glut_ = 52, 140, 97). ^∗∗^p < 0.01, Kruskal-Wallis ANOVA with Dunn’s multiple comparisons test. (F) Proportions of astrocytes sorted by response direction following acetylcholine, ATP, and glutamate application. (G) Average ΔF/F_0_ during drug application for glutamate plotted against ΔF/F_0_ for ATP shows most astrocytes that are excited by glutamate are also excited by ATP and vice versa. (H) Astrocytes are more likely to have matched responses between glutamate and ATP. Chi-square test between matched versus mismatched astrocytes. Fisher’s exact test matched versus mismatched p = 2.35e−11, OR = 3.24. (I) Venn diagram showing considerable overlap of matched responses of astrocytes to all three neurotransmitters. (J) Protocol for tetanus toxin (TetX) application. (K) Average traces for glutamate- and ATP-evoked excitatory and inhibitory responses with and without TetX. Line is smoothed average and shaded band SEM. (L) Blocking vesicular transmission does not suppress astrocyte responses to transmitter application (left to right, n_Pre-TetX__ATP_ = 21, 6; n_Post-TetX__ATP_ = 16, 35; n_Pre-TetX__Glut_ = 18, 11; n_Post-TetX__Glut_ = 14, 9). n.s. > 0.05 Kruskal-Wallis ANOVA with Dunn’s multiple comparisons test. Individual data points are single astrocytes across multiple animals. See also Figure S6.

Astrocytes have been proposed to facilitate fast synaptic activity by releasing gliotransmitters such as glutamate, ATP, and D-serine.^89^ In mammals, glutamate binding to mGluR5 triggers astrocytic D-serine release^90^ and the combination of glutamate and D-serine produces maximal NMDAR activation. We therefore tested whether fly astrocytes were responsive to glutamate application by recording *in vivo* astrocytic Ca^2+^ responses (*R86E01-GAL4*>*UAS-GCaMP7f*) (Figures 6A and 6B). We included 1 μM tetrodotoxin (TTX) in the bath to block voltage-gated sodium channels and thereby inhibit polysynaptic neurotransmission. Surprisingly, we could classify astrocytes within individual flies depending on whether bath-applied glutamate evoked an increase, decrease, or no change in GCaMP fluorescence (Figure 6B). We also tested for astrocytic responses to acetylcholine (ACh), the predominant excitatory transmitter in the fly brain, and ATP which triggers Ca^2+^ waves in mammalian astrocytes.^91^^,^^92^ Astrocytes also exhibited differential responses to ACh and ATP (Figures 6C–6F). Moreover, the same astrocytes often exhibited similar responses to the different neurotransmitters. For instance, a substantial proportion of astrocytes were activated both by glutamate and ATP (Figures 6G, 6H, S6A, and S6B), and most of these had matched responses to all three transmitters (Figure 6I).

We used TetX to eliminate all vesicular release and negate the possibility that evoking local transmission accounts for differential astrocytic responses (Figure S6C).^93^ Although there was no change in magnitude of astrocyte responses to either ATP or glutamate following 1 h TetX treatment, there was a slight reduction in percentage of ATP responsive astrocytes (Figures 6J–6L and S6F). We verified the effectiveness of TetX by demonstrating that a polysynaptic odor-evoked response in mushroom body output neuron (MBON)-γ5β′2a was eliminated (Figures S6D and S6E). Therefore, neurotransmitters evoke direct stereotyped activation or inhibition of astrocytes. Differential astrocytic responses to neuronal activation were reported in the larval VNC.^94^

### Water deprivation modulates functional properties of astrocytes

**Figure 7:**
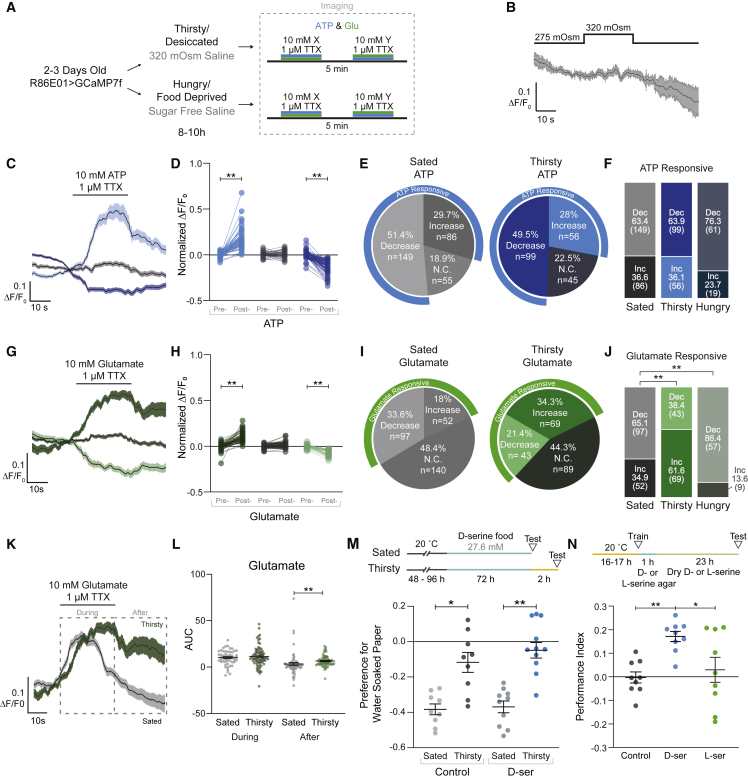
Water deprivation changes physiological properties of astrocytes (A) Protocol for water and food deprivation with calcium imaging experiments. (B) Acute exposure of high osmolarity saline does not induce a calcium response in astrocytes. (C) Average traces for ATP partitioned by response type in thirsty flies. Line is smoothed average and shaded band is SEM. (D) Responses pre- and post-treatment of ATP separated by categorical responses in water-deprived flies. ^∗∗^p < 0.01, Kruskal-Wallis ANOVA with Dunn’s multiple comparisons test. (E) Proportion of astrocyte responses to ATP application in sated and thirsty flies. (F) Astrocyte responses to ATP do not change with deprivation states. n.s. > 0.05 Fisher’s exact test with Bonferroni correction. (G) Average traces for glutamate partitioned by response type in thirsty flies. Line is smoothed average and shaded band SEM. (H) Responses pre- and post-treatment of glutamate separated by categorical responses in water-deprived flies. ^∗∗^p < 0.01, Kruskal-Wallis ANOVA with Dunn’s multiple comparisons test. (I) Proportions of astrocyte responses type to glutamate application in sated and thirsty flies. (J) Deprivation states differentially regulate astrocyte responsivity to glutamate application. The number of glutamate responsive astrocytes increases in thirsty flies and decreases in hungry flies. ^∗∗^p < 0.01, Fisher’s exact test with Bonferroni correction. (K) Excitatory astrocyte responses to glutamate application are prolonged in thirsty animals. (L) Area under the curve (AUC) of sections of traces marked in (K). ^∗∗^p < 0.01, Kruskal-Wallis ANOVA with Dunn’s multiple comparisons test. (M) Flies pre-fed D-serine do not show differences in innate water seeking in the T-maze. ^∗^p < 0.05, ^∗∗^p < 0.01, Kruskal-Wallis ANOVA with Dunn’s multiple comparisons test. (N) Flies fed D-serine between training and testing show increased water memory performance. ^∗^p < 0.05, ^∗∗^p < 0.01, Kruskal-Wallis ANOVA with Dunn’s multiple comparisons test. For (D), (H), and (L), individual data points are single astrocytes across multiple animals. See also Figure S7.

We next tested whether *in vivo* astrocytic responses to applied neurotransmitters changed in water- and food-deprived flies (Figure 7A). To prevent deprived flies from being “re-satiated” by application of normal extracellular saline, the saline composition was adjusted to reflect the desired physiological state of the animal.^19^^,^^95^ Astrocytes did not show a Ca^2+^ response to a brief increase in osmolarity (Figure 7B). Similar to sated flies, water- and food-deprived flies showed differential astrocytic responses to glutamate and ATP (Figures 7C–7J and S7A–S7C). However, only water-deprived flies showed an increase in the astrocyte proportion excited by glutamate, whereas ATP responsiveness remained the same (Figures 7F and 7J). Excitatory glutamate and ATP responses also appeared prolonged in thirsty flies compared with controls (Figures 7K, 7L, and S7D). Finally, thirsty flies had a higher proportion of mismatched astrocyte responses to ATP and glutamate, suggesting a loss of correlated responses (Figure S7G). In sum, water deprivation increases the proportion of glutamate-activated astrocytes and sustains glutamate-evoked Ca^2+^ responses.

### D-serine promotes water-memory expression

Prior work implicated specific neuronal pathways in regulating water consumption and thirst-dependent control of innate and learned water seeking.^19^, ^20^, ^21^, ^22^ Since astrocytes broadly innervate the neuropil, we tested whether D-serine promoted water vapor seeking and memory expression in water-sated flies, like it did for drinking. Interestingly, the same 3-day feeding of D-serine that increased drinking did not promote attraction to water vapor (Figure 7M). We next tested thirst-dependent expression of water memory performance. Water-deprived flies were trained to associate an odor with water reward, and after training, they were housed in vials with agarose for 1 h then dry vials containing either nothing, D-serine, or L-serine powder. Only flies fed with D-serine showed 24 h water memory performance (Figure 7N). These data demonstrate that dietary D-serine promotes the expression of water-seeking memory, in addition to drinking behavior.

## Discussion

### Single-cell RNA-seq reveals a glial response to water deprivation

Our scRNA-seq studies of thirst revealed astrocytes and other glia to be the principal transcriptionally responsive cells in the fly brain. We did not identify significant transcriptional changes within peptidergic^18^, ^19^, ^20^ or monoaminergic neurons,^21^^,^^22^^,^^96^ perhaps due to the relative scarcity of these neurons in our datasets. Although glial expression of many genes changed in thirsty flies, our initial loss-of-function RNAi screen only revealed potential roles for *aay*, *inos* (myo-inositol-1-phosphate synthase), and *Drat* in the control of drinking behavior. Results for *inos* and *Drat* were not corroborated by additional testing. Nevertheless, induction of myo-inositol synthesis and uptake is a known cellular response to osmotic stress.^97^ *Drat* might also be involved in resistance to stress of dehydration.^98^ We similarly speculate that other thirst-regulated genes maintain glial function under stress, without impacting fluid intake in a measurable way.

### Glial D-serine is required for motivated water-procuring behaviors

A key approach to verifying a role for D-serine in thirst was the ability to alter fly behavior by providing D-serine in the diet. Dietary D- but not L-serine restored the drinking defect of *aay*-deficient flies. Moreover, feeding D- but not L-serine to water-sated flies mimicked the behavioral effect of water-deprivation—promoting both drinking and water-seeking memory expression. We therefore conclude that gliotransmission of D-serine is an essential element of thirst-state behavioral modulation. Changes in hunger-directed feeding were not evident. At present we do not know if dietary D-serine floods the entire fly and bypasses the need for glial release. Interestingly, although our experiments demonstrated D-serine enantiomer specific *aay* rescue and gain-of-function effects on thirst-related behaviors, a previous study^70^ rescued a fly sleep defect with L- and D-serine feeding. However, rescue of sleep depended on intestinal serine racemase conversion of L- to D-serine. Since the authors also identified intestinal NMDAR1 expressing neurons,^70^ we speculate that L-serine conversion in the gut produces enough D-serine to alter sleep via that route, but it produces insufficient quantities to act in the brain.^99^

### Does water-deprivation prime astrocytes to release D-serine?

We noted that increased *aay* expression resulted in detection of a larger proportion of astrocytes (74% versus 56%) expressing *aay* following water deprivation (Figure S7E). This potential broadening of expression might indicate that water deprivation induces *aay* to meet a forthcoming demand for D-serine release and/ or to replenish it following release from these astrocytes. Interestingly, a similar number of astrocytes (34% versus 18%) also gained sensitivity to exogenously applied glutamate in thirsty flies. Although we do not know if the same astrocytes are involved, it seems plausible that thirst increases astrocytic expression of *aay* and the number of glutamate responsive astrocytes, so that glutamate-releasing neurons can evoke their D-serine release. Although loss-of-function experiments revealed the importance of astrocytic *aay*, induction also occurred in cortex, ensheathing, and surface glia. The role of *aay* in these other glial types is currently unclear. Elevated expression of *aay* was attenuated when flies were permitted to quench their thirst, consistent with induction serving an enhanced need for D-serine synthesis.

### Astrocytic D-serine facilitates the activity of glutamatergic circuits

We established that D-serine functions as an NMDAR co-agonist in *Drosophila*, like it does in mammals.^69^ Neuronal NMDA-evoked Ca^2+^ transients were potentiated by D-serine co-application and blocked by ketamine. Moreover, feeding D-serine did not promote drinking in flies harboring a K558Q point substitution in the NMDAR1 subunit that renders it insensitive to D-serine.^74^ Importantly, our ultrastructural analyses identified typical TPSs within the adult fly brain, as well as a new and more common anatomical motif where astrocytic processes engulf presynaptic boutons. Astrocytic processes are therefore well placed to potentially support synaptic transmission acting on either the pre- or post-synaptic neuron. Glial engulfment of presynaptic boutons may permit D-serine or other neuromodulators to regulate extra-synaptic NMDARs or other receptors to tune presynaptic transmitter release. Moreover, astrocytic processes in the SMP appear to be preferentially associated with glutamatergic synapses, despite cholinergic synapses being most abundant in this region. We therefore propose that thirst-regulated astrocyte-released D-serine facilitates NMDA-type currents so that the relevant glutamatergic synapses undergo short-term potentiation, allowing these water-procuring neural circuits to function more efficiently. If D-serine release depends on glutamate-evoked astrocytes at active TPSs, then its use will be need dependent and circuit specific. It is also conceivable that synaptic astrocyte processes can be remodeled by osmosensitive swelling and retraction.^100^ Further work is required to locate the critical D-serine modulated synaptic connections in the fly brain. We assume different circuits will underlie D-serine regulated learned water seeking and consumption.^19^, ^20^, ^21^, ^22^^,^^96^

### Do glia directly sense water deprivation?

Although glia are ideally positioned for both integrating hemolymph metabolic and osmotic states and instructing neuronal activity, we did not observe obvious astrocytic Ca^2+^ responses when the osmolarity of the extracellular solution was briefly increased. It is possible that astrocyte activation requires a longer and more sustained exposure to hyperosmotic solution, that they are instead sensitive to hypovolaemic changes, or that water deprivation does not induce an astrocytic Ca^2+^ response (we also do not know whether thirst-driven glial transcriptional responses are Ca^2+^-dependent). In addition, although fly perineurial and subperieurial glia provide the barrier between the hemolymph and brain, cortex glia wrap neuronal cell bodies, and astrocytes permeate the neuropil and associate with synaptic compartments. It is therefore plausible that the glia layers permit transduction of osmosensing signals to astrocyes. For example, Ca^2+^ oscillations in cortex glia, which require the Na^+^/Ca^2+^, K^+^ exchanger zydeco,^101^ could influence astrocyte activity/Ca^2+^, as they are directly connected.^102^

Astrocytic Na_(X)_ channels have been implicated in sodium sensing within the mouse SFO.^103^ Our scRNA-seq data reveal glial-specific expression of several potential osmo-sensing molecules. Solute carrier transporters such as *Ncc69*, *NKCC*, and *inebriated* (*ine*) localized to glial clusters (Data S2). Interestingly, the *ine*-encoded Na^+^/Cl^−^ dependent neurotransmitter transporter is required for water homeostasis in the Malpighian tubules.^104^ Additionally, *ine* negatively regulates neuronal activity, which could be a mechanism by which astrocytes couple osmotic regulation with neurotransmission.^105^ Several TRP genes encode mechanosensitive channels responsive to changes in cell volume.^106^ The *pain* and *wtrw* TRP genes were exclusively detected in glia (Data S2). Finally, glia exhibit restricted expression of the inwardly rectifying potassium channel genes *Irk2 and Irk3*, which are necessary for proper fluid excretion and renal function in *Drosophila*.^107^ Therefore, glia may sense osmotic changes and regulate osmotic balance in the brain.

### Thirst specificity

Although *Drosophila* are raised in the lab on food containing water, flies possess independent control mechanisms that permit selective food or water seeking.^20^, ^21^, ^22^^,^^96^^,^^108^ Thirst and hunger are however also coupled on a behavioral and neuronal level. For instance, prandial thirst is triggered by food ingestion to preemptively recalibrate blood osmolality.^4^^,^^16^^,^^19^^,^^109^ D-serine apparently distinguishes between water and food consumption. In addition, astrocytes showed opposing physiological changes with food compared with water deprivation (Figure 7J), and thirst-regulated genes barely changed when flies were hungry (Figure S2C). D-serine elevation, either by *aay* overexpression or D-serine feeding, promoted expression of water-seeking memory and water consummatory behaviors. Therefore, astrocytic *aay*-dependent D-serine signaling may provide a more general thirst modulation, as opposed to only regulating seeking^20^ or drinking.^19^ Further work is required to understand how thirst-driven D-serine interacts with other thirst-dependent modulatory signals. It will be interesting to determine whether D-serine influences sodium appetite and interacts with modulatory processes relevant to other motivational states.^22^^,^^95^^,^^110^, ^111^, ^112^, ^113^

### Could D-serine be a conserved state-mechanism in mammals?

Thirst alters brain-wide population dynamics in the mouse.^114^ Since astrocytes permeate all regions of the brain, it is possible that D-serine contributes to the broad thirst-driven modulation of neural activity. In addition, we note that a lower D-serine serum concentration has been linked to patients suffering from Schizophrenia,^115^ 6%–20% of whom are polydipsic.

## STAR★Methods

### Key resources table

**Table:** 

REAGENT or RESOURCE	SOURCE	IDENTIFIER
**Chemicals, peptides, and recombinant proteins**		

Drierite, 8 mesh	ACROS Organics / Fisher Scientfic	Cat#10185130
Schneider's culture medium	Gibco / Thermo Fisher Scientific	Cat#21720024
d(–)-2-amino-5-phosphonovaleric acid	Sigma-Aldrich	Cat#A8054
DNQX	Sigma-Aldrich	Cat#D0540
Tetrodotoxin	Abcam	Cat#ab120054
DPBS (calcium and magnesium free)	Gibco / Thermo Fisher Scientific	Cat#14190086
Papain	Sigma-Aldrich	Cat#P4762
Collagenase I	Sigma-Aldrich	Cat#C2674
DAPI	Thermo Fisher Scientific	Cat#D1306
BSA	New England Biolabs	Cat#B9000S
D-Serine	Tokyo Chemical Industry	Cat#S0033
L-Serine	Tokyo Chemical Industry	Cat#S0035
Glycine	Sigma-Aldrich	Cat#G8898
N-methyl-D-aspartic acid	Sigma-Aldrich	Cat#M3262
Acetylcholine	Sigma-Aldrich	Cat#A6625
Adenosine Tri-Pphosphate	Sigma-Aldrich	Cat#A2383
Glutamate	Sigma-Aldrich	Cat#G0355000
Adenosine	Sigma-Aldrich	Cat#A9251
Tetanus toxin	Sigma-Aldrich	Cat#T3194
α,β-methylene adenosine 5’-diphosphate	Sigma-Aldrich	Cat#M3763
4-Methylcyclohexanol (98%)	Sigma-Aldrich	Cat#153095
3-octanol (99%)	Sigma-Aldrich	Cat#218405

**Critical commercial assays**		

Chromium Single Cell 3’ Library & Gel Bead Kit v2, 4 rxns	10X Genomics	Cat#PN-120267
LightCycler 480 Probes Master	Roche	Cat#4887301001
RNeasy Mini Kit	Qiagen	Cat#74104
SuperScript III First-Strand Synthesis SuperMix	Invitrogen	Cat#18080400
Universal Probe Library	Roche	Cat#04683633001; Cat#04869877001
LightCycler 480 Probes Master	Roche	Cat#4887301001

**Deposited data**		

Raw sequencing data and processed dataset	This study	GEO: GSE207799
Drosophila melanogaster reference genome (dm6) and gene annotations (FlyBase, release 6.25, FB2018_06)	Thurmond et al.^116^	https://flybase.org/
Full Adult Female Brain (FAFB) transmission electron microscope (TEM) dataset	Zheng et al.^85^	https://temca2data.org/

**Experimental models: Organisms/strains**		

*Drosophila*: 0273-GAL4	Burke et al.^117^ and Gohl et al.^118^	N/A
*Drosophila*: w; repo-GAL4, tub-GAL80ts; repo-GAL4	S. Schirmeir & C. Klämbt	N/A
*Drosophila*: R86E01-GAL4	Bloomington Drosophila Stock Center; Kremer et al.^58^	RRID:BDSC_45914
*Drosophila*: R85G01-GAL4	Bloomington Drosophila Stock Center; Kremer et al.^58^	RRID:BDSC_40436
*Drosophila*: R66C08-GAL4	Bloomington Drosophila Stock Center	RRID:BDSC_49412
*Drosophila*: UAS-mCherry	NA	Lab stock
*Drosophila*: UAS-aay^RNAi^	Vienna Drosophila RNAi Center	RRID:FlyBase_FBst0454865
*Drosophila*: *Inos*^RNAi^	Vienna Drosophila RNAi Center	RRID:FlyBase_FBst0472636
*Drosophila*: UAS-*GstD1*^RNAi^	Vienna Drosophila RNAi Center	RRID:FlyBase_FBst0475105
*Drosophila*: UAS-*CG33970*^RNAi^	Vienna Drosophila RNAi Center	RRID:FlyBase_FBst0462630
*Drosophila*: UAS-*Socs36E*^RNAi^	Vienna Drosophila RNAi Center	RRID:FlyBase_FBst0469597
*Drosophila*: UAS-*Irc*^RNAi^	Vienna Drosophila RNAi Center	RRID:FlyBase_FBst0472971
*Drosophila*: UAS-*GstD9*^RNAi^	Vienna Drosophila RNAi Center	RRID:FlyBase_FBst0475656
*Drosophila*: UAS-Obp44a^RNAi^	Vienna Drosophila RNAi Center	RRID:FlyBase_FBst0464970
*Drosophila*: UAS-Sod3^RNAi^	Vienna Drosophila RNAi Center	RRID:FlyBase_FBst0471232
*Drosophila*: UAS-*CG9377*^RNAi^	Vienna Drosophila RNAi Center	RRID:FlyBase_FBst0464792
*Drosophila*: UAS-*Cyp28d1*^RNAi^	Vienna Drosophila RNAi Center	RRID:FlyBase_FBst0470866
*Drosophila*: UAS-*Pcyt2*^RNAi^	Vienna Drosophila RNAi Center	RRID:FlyBase_FBst0477620
*Drosophila*: UAS-Drat^RNAi^	Vienna Drosophila RNAi Center	RRID:FlyBase_FBst0480136
*Drosophila*: UAS-*CG33970* (*osy*)	Bernard Moussian; Wang et al.^119^	N/A
*Drosophila*: UAS-*aay*	FlyORF	RRID:FlyBase_FBst0501667
*Drosophila*: UAS-*GstD9*	FlyORF	F004171
*Drosophila*: UAS-*Obp44a*	FlyORF	F003929
*Drosophila*: UAS-*Sod3*	FlyORF	F003855
*Drosophila*: UAS-*Daao1*	Dai et al.^70^	N/A
*Drosophila*: UAS-*Shi*^ts1^	Kitamoto^120^	Lab stock
*Drosophila*: UAS-*Kir2.1*	Paradis et al.^121^	Lab stock
*Drosophila*: UAS-TNT E	Sweeney et al.^122^	Lab stock
*Drosophila*: UAS-GCamP7f	Dana et al.^123^	RRID:BDSC_79031; RRID:BDSC_80906
*Drosophila*: *NMDAR1*^K558Q^	Nigel Atkinson; Troutwine et al.^74^	N/A
*Drosophila*: *NMDAR1*^F654A^	Nigel Atkinson; Troutwine et al.^74^	N/A

**Oligonucleotides**		

TTCGCGAGGATGAATACGAT	This study	*SdhA* – FWD
CACGAGAGCGTGTGCTTG	This study	*SdhA* – REV
AAAAAGCTCCGGGAAAAGG	This study	*GAPDH* – FWD
AATTCCGATCTTCGACATGG	This study	*GAPDH* – REV
CACCATGAAGAACGCTGTTG	This study	*Obp44* – FWD
GCTTGTAGTCGGAGGCAGAG	This study	*Obp44* – REV
CCGTTTCCACGACATTGAGT	This study	*Drat* – FWD
GTTAATGCCTTGATGGGGAAC	This study	*Drat* – REV
TGGCTCCCTTTATCCCAAG	This study	*GstD9* – FWD
AAAAACAGGCGCTGATTGAT	This study	*GstD9* – REV
CGGTGACTCCCTTACCGTAG	This study	*GstD1* – FWD
TTTGGCCACCTCGAATGT	This study	*GstD1* – REV
AAAAAGCCAGCAAACCAAAA	This study	*Socs36E* – FWD
AGGTGATGACCCATTGGAAG	This study	*Socs36E* – REV
TGCAATGGGTGGAATTCAG	This study	*Irc* – FWD
ACCATTTCGAAGCAGGAATC	This study	*Irc* – REV
CCCCTGAAAAACGTCTATGC	This study	*aay* – FWD
AGCTATCGTATTCGCCCAAA	This study	*aay* – REV
AGTGCTGTAATATCCCCGATAAAC	This study	*CG9377* – FWD
CAGAGATCATGGCGTCCTC	This study	*CG9377* – REV
ACCGCTCTTTATGGACTTTGAG	This study	*Pcyt2* – FWD
GCATGGCCTTGTCATCGTA	This study	*Pcyt2* – REV

**Software and algorithms**		

CellRanger	10X Genomics	https://support.10xgenomics.com/single-cell-gene-expression/software/overview/welcome; RRID:SCR_017344
R	R Development Core Team, 2008	http://www.R-project.org/; RRID:SCR_001905
Seurat v3	Hafemeister and Satija^124^ and Stuart et al.^125^	https://satijalab.org/seurat/; RRID:SCR_016341
DoubletFinder	McGinnis et al.^126^	https://github.com/chris-mcginnis-ucsf/DoubletFinder; RRID:SCR_018771
ZINB-WaVE	Risso et al.^46^ and Van den Berge et al.^47^	https://github.com/drisso/zinbwave
DESeq2	Love et al.^48^	https://bioconductor.org/packages/release/bioc/html/DESeq2.html; RRID:SCR_015687
edgeR	Robinson et al.^49^ and McCarthy et al.^127^	https://bioconductor.org/packages/release/bioc/html/edgeR.html; RRID:SCR_012802
ClusterProfiler	Yu et al.^128^	https://github.com/YuLab-SMU/clusterProfiler
GOplot	Walter et al.^129^	https://wencke.github.io/
ScanImage 3.8	Pologruto et al.^130^	http://scanimage.vidriotechnologies.com
Fiji	NIH; Schindelin et al.^131^	https://fiji.sc/
FlyWire	Dorkenwald et al.^84^	https://flywire.ai/
Neuroglancer	Dorkenwald et al.^84^	https://github.com/google/neuroglancer
CAVEclient		https://github.com/seung-lab/CAVEclient
FAFBseg v.1.4		https://github.com/navis-org/fafbseg-py
Navis v.0.6		https://github.com/navis-org/navis
trimesh		https://github.com/mikedh/trimesh
skeletor 1.1	Au et al.^132^	https://github.com/navis-org/skeletor
Blender 3.0	Blender Community	https://www.blender.org/download/releases/3-0/
GraphPad Prism 9	GraphPad Software	https://www.graphpad.com/scientific-software/prism/; RRID:SCR_002798
Adobe Illustrator	Adobe Systems	https://www.adobe.com/uk/products/illustrator.html; RRID:SCR_010279

**Other**		

10 mm CellTrix strainer	Sysmex	Cat#04-0042-2314
Fuchs-Rosenthal haemocytometer	VWR	Cat#631–1096
Fisherbrand chromatography paper 180g/m^2^ 460x570 mm	Fisher Scientific	Cat#15649494

### Resource availability

#### Lead contact

Further information and requests for resources and reagents should be directed to and will be fulfilled by the lead contact, Scott Waddell (scott.waddell@cncb.ox.ac.uk).

Correspondence regarding the single-cell transcriptomic data and its analysis should be addressed to Vincent Croset (vincent.croset@durham.ac.uk).

#### Material availability

This study did not generate new unique reagents.

### Experimental model and subject details

#### Drosophila strains

GAL4 drivers used in this study are 0273-GAL4,^117^^,^^118^ R66C08-GAL4 (MBON-γ5β′2a),^133^ repo-GAL4, tub-GAL80ts; repo-GAL4,^56^^,^^134^^,^^135^ R86E01-GAL4^58^ and R85G01-GAL4.^58^ UAS lines are w; +; UAS-mCherry, UAS-*aay*^RNAi^ (VDRC, 23179), UAS-*Inos*^RNAi^ (VDRC, 100763), UAS-*GstD1*^RNAi^ (VDRC, 103246), UAS-*CG33970*^RNAi^ (VDRC, 38661), UAS-*Socs36E*^RNAi^ (VDRC, 51821), UAS-*Irc*^RNAi^ (VDRC, 101098), UAS-*GstD9*^RNAi^ (VDRC, 103798), UAS-*Obp44a*^RNAi^ (VDRC, 43203), UAS-*Sod3*^RNAi^ (VDRC, 8760), UAS-*CG9377*^RNAi^ (VDRC, 42835), UAS-*Cyp28d1*^RNAi^ (VDRC, 7870), UAS-*Pcyt2*^RNAi^ (VDRC, 105794), UAS-*Drat*^RNAi^ (VDRC, 108325), UAS-*osy*,^119^ UAS-*GstD9* (FlyORF, F004171), UAS-*Obp44a* (FlyORF, F003929), UAS-*Sod3* (FlyORF, F003855), UAS-*aay* (FlyORF, F002296), UAS-*CG12338*,^70^ UAS-*Shi*^ts1^,^120^ UAS-*Kir2.1*,^121^ UAS-TNT E,^122^ UAS-*GCaMP-7f*.^123^ Mutant strains are *NMDAR1*^K558Q^ and *NMDAR1*^F654A^.^74^ Flies were raised on standard cornmeal food under a 12:12 light:dark cycle at 60% humidity and 25°C, unless otherwise stated. 3-day old mixed sex flies were used for single-cell transcriptomics, 3-6 day old mixed sex flies for water preference, RT-qPCR and imaging experiments, and 3-8 day old male flies for water consumption tests.

### Method details

#### Water preference assays

Dehydration and water preference assays were performed as described.^21^ Briefly, groups of 50-100 flies were stored for a given time period in vials containing a ∼2 cm layer of Drierite topped with a piece of cotton and a dried sucrose-coated filter paper. Vials were kept in a sealed container with a layer of Drierite at the bottom. Flies were (re-)hydrated flies in vials containing 1% agarose and a wet piece of sucrose-coated filter paper. After the appropriate water-deprivation period, flies were transferred into a T-maze and given the choice between two chambers lined with either a dried or a wet piece of filter paper. Preference Index was calculated as the number of flies in the wet tube minus the number of flies in the dry tube, divided by the total number of flies in each experiment.

#### Brain dissociation and cell collection

Brains (*0273-Gal4>mCherry*) were dissected and dissociated as described.^33^ Briefly, 24 central brains from an equal number of male and female flies were individually dissected in ice-cold DPBS (Gibco, 14190–086) and immediately transferred into 1 mL toxin-supplemented Schneider’s medium (tSM: Gibco, 21720–001 + 50 mM d(-)-2- amino-5-phosphonovaleric acid, 20 mM 6,7-dinitroquinoxaline-2,3-dione and 0.1 mM tetrodotoxin) on ice. Brains were washed once with 1 mL tSM and incubated in tSM containing 1.11 mg/mL papain (Sigma, P4762) and 1.11 mg/mL collagenase I (Sigma, C2674). Brains were washed once more with tSM and subsequently triturated with flame-rounded 200 mL pipette tips. Dissociated brains were resuspended in 1 mL PBS + 0.01% BSA and filtered through a 10 mm CellTrix strainer (Sysmex, 04-0042-2314). Cell concentration was measured using a disposable Fuchs-Rosenthal haemocytometer (VWR, 631–1096) under a Leica DMIL LED Fluo microscope. A typical preparation from 24 brains yielded ∼600,000 cells.

#### Library preparation, sequencing, and processing

mRNA barcoding was performed using the Chromium Single Cell 3′ Reagent Kit v3 (10x Genomics), following the manufacturer’s instructions. For each sample, we targeted an 8,000 cell recovery. Two libraries were prepared for each condition (sated, 6h dehydrated, 12h dehydrated and 12h dehydrated + 45min rehydrated). Libraries were sequenced with NovaSeq 6000 (Illumina) at Oxford’s Wellcome Trust Centre for Human Genetics. We obtained 3.419 billion reads, and used CellRanger 3.1.0 to map these to the FB2018_06 *Drosophila melanogaster* genome assembly (v6.25),^116^ and create digital gene expression (DGE) matrices.

#### Filtering and doublet removal

Cell barcodes with <300 or >4,500 features, >20,000 UMIs, >15% mitochondrial RNA, >10% rRNA or >15% ribosomal proteins were discarded. DGE matrices were then merged by condition, normalized, and scaled using SCTransform in Seurat v3.^124^^,^^125^ This included regression for the effects replicate and sex – based on expression of the male-specific lncRNA *roX1*.^136^ DGE matrices were integrated using CCA anchor-based methods, and cells were clustered using the Louvain algorithm on the shared nearest neighbor graph with a resolution of 2 and an UMAP reduction performed for visualization, using the top 20 Principal Components (PCs). Doublets were removed using a hybrid method. First, DoubletFinder^126^ was run on data processed with the standard Seurat scaling method (without SCTransform), as we found that DoubletFinder failed to produce reliable BCmvn curves on SCtransformed data. This identified 3,493 potential doublets. Second, because the glial marker *nrv2*^137^ and markers for neurons releasing fast-acting neurotransmitters *VAChT*, *VGlut* or *Gad1* are normally not co-expressed, we considered it parsimonious to classify “cells” expressing two or more of these genes as doublets. So as not to needlessly discard cells containing small contamination from these genes, we set thresholds of 3 (*nrv2*), 1.5 (*VAChT*), 2.1 (*VGlut*) and 2.3 (*Gad1*), above which we considered these genes to be highly expressed. These values were estimated from the normalized and scaled expression levels as the local minima in each gene’s bimodal distribution of non-zero values. We used the same principle to detect doublets in Kenyon Cells. We first identified Kenyon Cell clusters based on expression of *Dop1R1*, *ey* and *mub*.^33^ We then flagged all cells co-expressing markers for more than one Kenyon Cell subtype, namely *Ca-alpha1T* for αβ, *ab* for γ and *CG8641* for α′β′,^33^ with thresholds of 1.5, 1.5 and 2.2, respectively (again estimated as the local minima in each gene’s bimodal distribution of non-zero values in their normalized and scaled expression levels). Overall, this co-expression strategy identified another 2,828 doublets. Doublets were evenly spread across clusters, with only six clusters comprised of >20% doublets. All doublets identified with either of these two methods (9.05% of all cells) were removed prior to subsequent analyses (Figure S1A).

#### Clustering

We developed a 4-step clustering pipeline with the aim of minimizing the number of PCs used for clustering and reducing the ‘curse of high dimensionality’ effects on nearest neighbor search.^138^^,^^139^ After doublet removal, data was clustered using the Louvain algorithm with the first 20 PCs and a resolution of 2 and an UMAP plot constructed using the same PC dimensions for visualization. Then each cluster was assigned to one of six major cell types present in the *Drosophila* brain, using expression of the following markers: *VAChT* (cholinergic), *VGlut* (glutamatergic), *Gad1* (GABAergic), *ey*, *Dop1R2*, *Pka-C1*, *mub* (Kenyon Cells), *Vmat* (monoaminergic) and *CG10433* (glia & astrocytes),^33^ with a seventh group containing non-assigned cells (“other”). Cells from each group were subsequently isolated. Data was normalized and scaled using SCTransform, and clustered and visualized on a UMAP plot, with 19, 17, 16, 12, 16, 14 and 12 PCs for each group, respectively. A resolution of 1 was used, except for the monoaminergic (4) and other (2) groups, with the aim of slightly over-clustering. Lastly, for each group a phylogenetic tree of all clusters was constructed in PCA space using Seurat’s BuildClusterTree function. Clusters on neighboring branches with less than 10 protein-coding genes differently expressed between them (Wilcoxon signed-rank test, adjusted p<0.05) were fused.

#### Annotation

Specific clusters were annotated based on the expression of the following known marker genes. Olfactory projections neurons (cholinergic): *acj6*, *ct*, *Lim1*,^140^, ^141^, ^142^ ellipsoid body (EB) large-field ring neurons (GABAergic): *cv-c*, *Dh31*, *Octbeta2R*, *5-HT7*,^143^, ^144^, ^145^, ^146^ EB small-field ring neurons (GABAergic): *cv-c*, *Dh31*, *Octbeta2R*, not *5-HT7*, ventral and dorsal fan-shaped body (GABAergic): *cv-c*, *Dh31, sNPF*, not *Octbeta2R*,^147^ medial fan-shaped body: *cv-c*, *Dh31*, not sNPF, not *Octbeta2R*, a/b Kenyon Cells: *sNPF*, *Eip93F*,^33^^,^^148^ g Kenyon Cells: *sNPF*, *ab*,^33^ a′/b′ Kenyon Cells: *CG8641*,^33^ dopaminergic neurons (monoaminergic): *ple*, *DAT*,^149^^,^^150^ serotonergic neurons (monoaminergic): *SerT*, *Trh*,^151^^,^^152^ octopaminergic neurons (monoaminergic): *Tdc2*, *Tbh*,^153^^,^^154^ tyraminergic neurons (monoaminergic): *Tdc2*, not *Tbh*, astrocytes: *AANAT1*, *alrm*, *Gat*, *e*,^155^, ^156^, ^157^ surface glia: *Tret1-1*, *Mdr65*,^32^^,^^158^ ensheathing glia: *zyd*, *trol*,^35^^,^^101^ cortex glia: *zyd*, *wrapper*.^35^ Thresholds for each gene were set manually. Top markers for each cluster were calculated (Data S1).

#### Differential expression (DE)

To account for zero-inflation triggered by dropout events and enable the use of DE tools initially created for analyzing bulk RNA-sequencing data, we generated cell-specific weights, using ZINB-WaVE.^46^^,^^47^ Both DESeq2^48^ and edgeR^49^^,^^127^ were then used to calculate differential expression between pairs of conditions. Genes with |log_2_(FC)|>1 and adjusted p-value<0.05 calculated with either method were considered to be differentially expressed.

#### Pathway analysis

Gene Ontology Biological process over-representation test was performed for the differentially expressed genes shown in Figure 2E, using the Bioconductor package ClusterProfiler.^128^ Significantly enriched GO terms based on p-values were then visualized as a chord diagram with GOplot.^129^

#### RT-qPCR

Quantitative PCR experiments were performed as described.^33^ In brief, total RNA was extracted from groups of 40 fly heads using the RNeasy Mini kit (Qiagen 74104). mRNA was then reverse-transcribed using the SuperScript III First-Strand Synthesis SuperMix (Invitrogen, 18080400) according to manufacturer’s instructions. qPCR was performed in a Light- Cycler 480 Instrument II (Roche, 05015243001) using the Universal Probe Library system (UPL; Roche, 04683633001 and 04869877001). Each 10 mL reaction contained 2.4 mL of pre-amplified cDNA, 0.4 mM of each primer (designed with Roche Assay Design Center), 0.2 mM of UPL probe, and 5 mL LightCycler 480 Probes Master (Roche, 4887301001). Cycles were as follows: 95°C, 10′; 45x [95°C, 10′; 60°C, 30′; Fluorescence acquisition; 72°C, 1′].

#### D-serine Feeding

For D-serine feeding 2.9 g/L or 27.6 mM of D-serine (S0033 Tokyo Chemical Industry) was mixed with 2% agarose and 5% sucrose. We used the same concentration of L-serine (S0035 Tokyo Chemical Industry). For control treated groups we used 2% agar and 5% sucrose.

#### CAFE Assay

CAFE assay was performed as described^57^ but adjusted for water consumption. Groups of 12 male flies were first dehydrated for 1 h in vials containing Drierite without indicator (ACROS Organics). Flies were then transferred to CAFE vials containing four glass capillaries loaded with 5 μL of deionized water and ∼0.1 μL of mineral oil. Vials were loaded into a tray covered with plastic wrap to reduce water loss due to evaporation. We also used 3 empty CAFE vials to assess the level of evaporation to subtract from the amount of water consumed by the flies. Flies were kept in either 20°C or 30°C incubators. Food consumption CAFE used 5% yeast extract and 5% sucrose solution and 10 male flies per vial measuring consumption across 24 h. Vials also contained 10 mL of 1% agarose. We kept track of the number of deaths for each vial and re-adjusted consumption values based on the number of flies alive by the conclusion of the assay. Water and food consumption in μL is recorded as total water consumption of 12 flies minus the average evaporation volume from the empty vials.

D-serine preference CAFE involved storing groups of 8 male flies per vial at 30°C for 18–20 h prior to the start of the assay. We then transferred the flies into CAFE preference vials containing two capillary tubes loaded with normal 5% yeast extract and 5% sucrose and two capillary tubes loaded with 2.9 g/L (27.6 mM) of D-serine (S0033 Tokyo Chemical Industry) with 5% yeast extract and 5% sucrose. Vials also contained 10 mL of 1% agarose. Flies were then given 24 h to feed then were removed. D-serine Preference Index was calculated as the volume of D-serine food minus volume of normal food divided by the total volume of food consumed.

#### Manual Feeding Assay

The water feeding assay was performed as described^19^ with minor adjustments. Flies were briefly anesthetized on ice and fixed to a glass slide using beeswax. The slide was then transferred to an empty pipette tip box containing Drierite without indicator (ACROS Organics), closed, then wrapped with parafilm at the seam. For experiments performed at 30°C we put the flies in an incubator for the 2 h dehydration step then performed the experiment in a temperature (30°C) and humidity-controlled (60-65%) booth. The flies were hand fed with a syringe containing deionized water. If the experiments were performed at 30°C the water was warmed to minimize temperature changes in the fly during feeding. We presented the water 10 times to each fly and measured cumulative time spent drinking the water. Presentations were made such that we briefly touched the fly’s legs with the water. Flies were excluded if they exhibited little to no movement when touched on their abdomen with the needle. For NMDAR single-site mutant flies we used a 2.5 h dehydration step instead of 2 h as the background Canton-S strain from the Atkinson lab (UT, Austin) showed greater dehydration resistance than Canton-S strain of the Waddell lab.

#### Hygrosensation/Water memory in the T-maze

We used 3-5 day old flies to assess hygrosensation in the water T-maze, as described.^39^ Briefly, 24 h prior to testing we group housed ∼100 flies per vial on standard cornmeal/agar food and a 20x60 mm piece of filter paper. If flies were water deprived, they were housed in vials containing desiccant for 2 h before testing. Testing was performed in a temperature (30°C) and humidity-controlled (60-65%) booth. Flies were loaded into a T-maze and given a 2 min choice between an arm containing wet filter paper or dry filter paper. Preference Index was calculated as the number of flies in the wet arm minus the number in the dry arm, divided by the total number of flies.

For water memory assays we used 3-5 day old flies. Prior to training, flies were water deprived for 16-17 h in vials containing dessicant. Water-deprived flies were trained to associate odor with water reward^159^ then were immediately transferred to vials containing 1% agarose (as a water source) supplemented with nothing, D-serine, or L-serine, 29 g/L (276mM). They were then housed for an additional 23 h in vials containing dry sucrose, dry sucrose and D-serine, or dry sucrose and L-serine, before being tested in the T-maze for water memory performance.

#### Two-Photon Calcium Imaging

Imaging experiments were performed using 3-7 day old flies as described previously.^160^^,^^161^ Briefly, flies were immobilized by cooling on ice and mounted in a custom-built chamber to allow free movement of their legs. For control conditions we used an external saline containing 103 mM NaCl, 3 mM KCl, 5 mM N-Tris, 10 mM trehalose, 10 mM glucose, 7 mM sucrose, 26 mM NaHCO_3_, 1 mM NaH_2_PO_4_, 1.5 mM CaCl_2_, 4 mM MgCl_2_, osmolarity 275 mOsm, pH 7.3. The head capsule was opened under room temperature carbogenated (95% O_2_, 5% CO_2_) external saline. The mounted fly was placed under the two-photon microscope (Scientifica). We used a Ti-Sapphire laser (Chameleon Ultra II, Coherent) to excite fluorescence using 140 fs pulses with 80 MHz repetition rate at 910 nm. We acquired 256 x 256 pixel images at 5.92 Hz controlled by ScanImage 3.8 software.^130^

For analysis, the two-photon images were manually segmented using Fiji.^131^ With a custom Fiji script we measured the average baseline fluorescence (F_0_) for 14 s prior to each drug treatment or stimulus delivery. F/F_0_ describes the fluorescence relative to the baseline. For drug delivery treatments we defined the “Pre-” treatment as the average F/F_0_ value for 14 s prior to the drug delivery and the “Post-” treatment as the average F/F_0_ in the 25 s from onset of drug delivery to the offset. To account for inter-cell baseline differences in F/F_0_ we normalized the “Pre-” treatment to equal 0 for each cell. For area under the curve (AUC) calculation we measured the approximated integral of F/F_0_ during the drug treatment (“During”) as well as after the treatment until the recording ended (“After”). To account for variance between individual cells, we normalized the beginning of the trace starting from the onset of drug delivery to equal 0 for each cell. For odor delivery AUC analysis we measured the F/F_0_ during the 5s odor presentation.

To image fly brains following desiccation or starvation, we starved and water deprived flies for 8-10 h prior to recordings. All recordings were performed between ZT 2-6 and deprived flies at ZT 18-22. Dehydration was performed as above.

#### Solutions

For acute drug application, we used a perfusion pump system (Fisher Scientific US 14-284-201) to continuously deliver saline at a rate of ∼0.043 mL/sec. All drugs were applied with in the presence of 1μM tetrodotoxin (TTX) to block voltage-gated sodium channels and propagation of action potentials.

#### NR1+ neuron and astrocyte recordings

Recordings of NR1+ neurons (*nmdar1*-*KIGAL4*>*GCaMP7f*) were performed using either 4 mM Mg^2+^ or 0 mM Mg^2+^ saline. To compensate for the change in osmolarity with Mg^2+^ free saline, we added 1.5 mM CaCl_2_ and 2.5 mM NaCl. We used 20 mM N-methyl-D-aspartic acid (Sigma-Aldrich M3262), D-serine (Tokyo Chemical Industry - S0033), L-serine (Tokyo Chemical Industry – S0035), and glycine (Sigma-Adlrich G8898). Drug mixtures were maintained at room temperature prior to application. Before recordings we pre-loaded the pump with 1 mL of saline then 1 mL of drug mixture and allowed the pump to run continuously throughout the recording so there were no discontinuities in flow during recording. We flushed the pump for 5 min with saline between each drug application. When multiple drug treatments were performed the application order was randomized for each animal to account for sensitization/desensitization effects. To block NMDARs, we applied 15 mM ketamine with NMDA, D-serine, and TTX.

Astrocyte recordings (*R86E01-GAL4*>*GCaMP7f*) were performed using normal external saline, hyperosmotic 320 mOsm saline,^19^ and sugar free saline.^95^ Hyperosmotic saline was made by adjusting the total amount of added solute in our original saline recipe, while maintaining the same ratios, so that the osmolarity was increased to 320 mOsm. We applied 10 mM acetylcholine (Sigma-Aldrich A6625), 10 mM ATP (Sigma-Aldrich A2383), 10 mM glutamate (Sigma-Aldrich G0355000), or 10 mM adenosine (Sigma-Aldrich A9251) with 1 μM TTX (Sigma-Aldrich T3194). When multiple drug treatments were performed the application order was randomized for each animal.

For MBON-γ5β′2a (M6) recordings (*R66C08-GAL4*>*GCaMP7f*), flies were exposed to a constant air stream containing mineral oil solvent (air). Flies were sequentially exposed to 3-octanol (OCT) and 4-methylcyclohexanol (MCH) for 5 s with 30 s inter-odor interval. To measure dendritic responses of MBON-γβ′2a signals were simultaneously acquired from both hemispheres and average responses were analyzed.

For tetanus toxin (TetX) application, we replaced the bath saline with saline containing 1 μM TetX and incubated the flies for 1 h at room temperature. Halfway through the incubation the bath solution was mixed 4X by pipetting.

For α,β-methylene adenosine 5’-diphosphate (AMPCP) (Sigma-Aldrich M3763) application we incubated the flies with 100 μM AMPCP in normal saline for at least 30 min. Flies were then imaged using saline containing AMPCP and ATP.

#### Identification of astrocytes in EM reconstructions

We used FlyWire (www.flywire.ai)^84^ to construct the morphology of astrocytes in the Full Adult Female Brain (FAFB) transmission electron microscope (TEM) dataset.^85^ Astrocytes were identified and distinguished from other glia in the raw EM data by first following cell processes that cross the ensheathing glial boundary, and following them to find their cell body. Astrocytic morphology is characteristically distinct from that of neuronal soma tracts, and ensheathing or cortex glia.

In FlyWire, 3D meshes from automatic A.I. based^162^ reconstructions of neurons and glia are readily available, but manual expert review is required to add missing, or remove extraneous, branches. Following revision neurons and glial cells had no false continuations, by standard criteria that are previously described.^84^^,^^163^^,^^164^ In brief, an expert reviewer (>1000 h experience) first inspected the astrocytes for false continuations, and obvious missing branches. Then a methodical visual inspection of all branches with neuroglancer^84^ allowed the user to identify and correct further smaller errors. A second expert reviewer then performed the same review to generate a consensus. In this study, 4 astrocytes were submitted for extensive review to two experienced reviewers following every process section by section from the tip inward toward. This review process only resulted in the correction of a few smaller branches with no more than 2 connections each.

#### Classification of astrocytic processes

Tripartite synapses (TPS) contain presynaptic, postsynaptic, and astrocytic process, with all compartments making direct contact with the synaptic cleft. We found that astrocytic processes sometimes also cover a large part of the perimeter of a presynaptic bouton without contacting the synaptic cleft – a motif we classified as ‘engulfing’. Lastly astrocytic processes were often also found to contact the post-synaptic neuron below the synaptic cleft (within 500 nm of a presynaptic terminal. These contacts to post-synaptic neurons were not included in the TPS analyses.

#### Analysis of astrocyte-neuronal type relationship

3D meshes of cells in FlyWire, information about synapses with pre and postsynaptic partners,^165^ and neurotransmitter predictions^88^ were downloaded with the CAVEclient (github.com/seung-lab/CAVEclient) and further analysed using functionalities from FAFBseg v.1.4 (https://github.com/navis-org/fafbseg-py) and Navis v.0.6 (https://github.com/navis-org/navis) and custom scripts (available upon request). Synapses with CleftScore ≥ 50 and ConnectionScore ≥ 33^165^ were considered for analyses, after thresholds were manually reviewed in a sample of 500. Synapses were classified as TPS, when an astrocyte was found as a postsynaptic partner to a neuronal presynapse.

To determine the vicinity profile of an astrocyte, it’s mesh was obtained and skeletonised with trimesh v.3.9.32 (https://github.com/mikedh/trimesh) and Skeletor 1.1.^132^

To exclude somata and main branches we only considered nodes with a radius of < 300 nm. To generate the vicinity profile, synapses within a 2 μm bounding box around the nodes were filtered for the above criteria and classified by neurotransmitter usage. Then the histogram of distances between the nodes and synapses of each neurotransmitter was generated and gaussian kernel density estimates and bootstrap samples of the means of the distance distributions computed with Python base functions for statistical analyses (Code available on request).

#### 3D representations

3D representations and animations of astrocytes were created with Blender 3.0 (Blender Community, 2018) from meshes obtained from Flywire, via CAVEclient and neuroglancer within Flywire.

### Quantification and statistical analysis

#### Single-cell transcriptomics

Statistical methods employed for single-cell analysis are described above. In brief, Wilcoxon signed-rank test was used for marker analysis and cluster annotation. For differential expression, the Wald test implemented in DESeq2 and the binomial generalized log-linear model implemented in edgeR’s *glmWeightedF* function were used. For these tests, N represents the number of cells in relevant clusters, and significance was defined using an adjusted p-value<0.05 after Benjamini-Hochberg correction. When relevant, details are found in the figure legends.

#### Behavioral Statistical Analysis

All behavioral data was analyzed using Prism GraphPad software. Data were analyzed for normality using the Shapiro-Wilk test. Depending on normality we used a one-way ANOVA with Dunnett’s multiple comparisons or Kruskal-Wallis ANOVA with Dunn’s multiple comparisons. Details on statistical analyses and the N for each experiment can be found in the figure legends. Significance was defined using an alpha < 0.05.

#### Two-Photon Calcium Imaging Analysis

For analysis, the two-photon images were manually segmented using Fiji and analyzed using a custom MATLAB script.^131^ With a custom Fiji script we measured the average baseline fluorescence (F_0_) for 14 s prior to each drug treatment or stimulus delivery. F/F_0_ describes the fluorescence relative to the baseline. For drug delivery treatments we defined the “Pre-” treatment as the average F/F_0_ value for 14 s prior to the drug delivery and the “Post-” treatment as the average F/F_0_ in the 25 s from onset of drug delivery to the offset. To account for inter-cell baseline differences in F/F_0_ we normalized the “Pre-” treatment to equal 0 for each cell. For area under the curve (AUC) calculation we measured the approximated integral of F/F_0_ during the drug treatment (“During”) as well as after the treatment until the recording ended (“After”). To account for variance between individual cells, we normalized the beginning of the trace starting from the onset of drug delivery to equal 0 for each cell. For odor delivery AUC analysis we measured the F/F_0_ during the 5s odor presentation.

Data were analyzed for normality using the Shapiro-Wilk test. Depending on normality we used a one-way ANOVA with Dunnett’s multiple comparisons or Kruskal-Wallis ANOVA with Dunn’s multiple comparisons. Details on statistical analyses and the N for each experiment can be found in the figure legends. Significance was defined using an alpha < 0.05. For categorical data statistical significance was determined using a chi-square test.

#### Analysis of astrocyte-neuronal type relationship

3D meshes of cells in FlyWire, information about synapses with pre and postsynaptic partners,^165^ and neurotransmitter predictions^88^ were downloaded with the CAVEclient (github.com/seung-lab/CAVEclient) and further analysed using functionalities from FAFBseg v.1.4 (https://github.com/navis-org/fafbseg-py) and Navis v.0.6 (https://github.com/navis-org/navis) and custom scripts (available upon request).

Synapses with CleftScore ≥ 50 and ConnectionScore ≥ 33^165^ were considered for analyses, after thresholds were manually reviewed in a sample of 500. Synapses were classified as TPS, when an astrocyte was found as a postsynaptic partner to a neuronal presynapse.

To determine the vicinity profile of an astrocyte, it’s mesh was obtained and skeletonised with trimesh v.3.9.32 (https://github.com/mikedh/trimesh) and Skeletor 1.1.^132^

To exclude somata and main branches we only considered nodes with a radius < 300 nm. To generate the vicinity profile, synapses within a 2 μm bounding box around the nodes were filtered for the above criteria and classified by neurotransmitter usage. Then histograms of distances between the nodes and synapses of each neurotransmitter were generated. For shortest distances between all astrocytes and synapses of specific neurotransmitters probability densities and their functions from gaussian kernel density estimates were created for data representation in Figure 5J (matplotlib.pyplot.hist(density = True); scipy.stats.gaussian_kde(), with bandwidth method = Scott’s rule (default) and equal weighting). For testing of statistical significance the distributions of means from 10000 samples of the shortest distances between all astrocytes and synapses using specific neurotransmitters obtained via bootstrapping (numpy.random.choice() for the all 3 astrocytes and all synapses) (shown in Figure S5N) were tested by a two sided Welch’s t-test assuming non equal variance (scipy.stats.ttest_ind(equal_var = False)) significance was determined when p < 0.05 (Code available on request).

## Data Availability

Single-cell RNA-seq data have been deposited at GEO and are publicly available as of the date of publication. Accession numbers are listed in the key resources table. Detailed code for scSeq analyses is available at https://github.com/sims-lab/FlyThirst. Any additional information required to reanalyze the data reported in this paper is available from the lead contact upon request. All data reported in this paper will be shared by the lead contact upon request.

## Supplemental Information

Document S1. Figures S1–S7

Data S1. Top marker genes for each cluster in the entire data set, related to Figures 1 and S1

Data S2. Top markers for glial clusters, related to Figures 1 and 2

Document S2. Article plus supplemental information
