# Supplementary material for: Gliotransmission of D-serine promotes thirst-directed behaviors in Drosophila
Source: Curr Biol. 2022 Sep 26;32(18):3952–3970.e8. doi: 10.1016/j.cub.2022.07.038 (PMC9616736; doi:10.1016/j.cub.2022.07.038)
Supplement: Document S1. Figures S1–S7 [file mmc1.pdf]

Current Biology, Volume *32*

Supplemental Information

Gliotransmission of D-serine promotes thirst-directed behaviors in *Drosophila*

Annie Park, Vincent Croset, Nils Otto, Devika Agarwal, Christoph D. Treiber, Eleonora Meschi, David Sims, and Scott Waddell

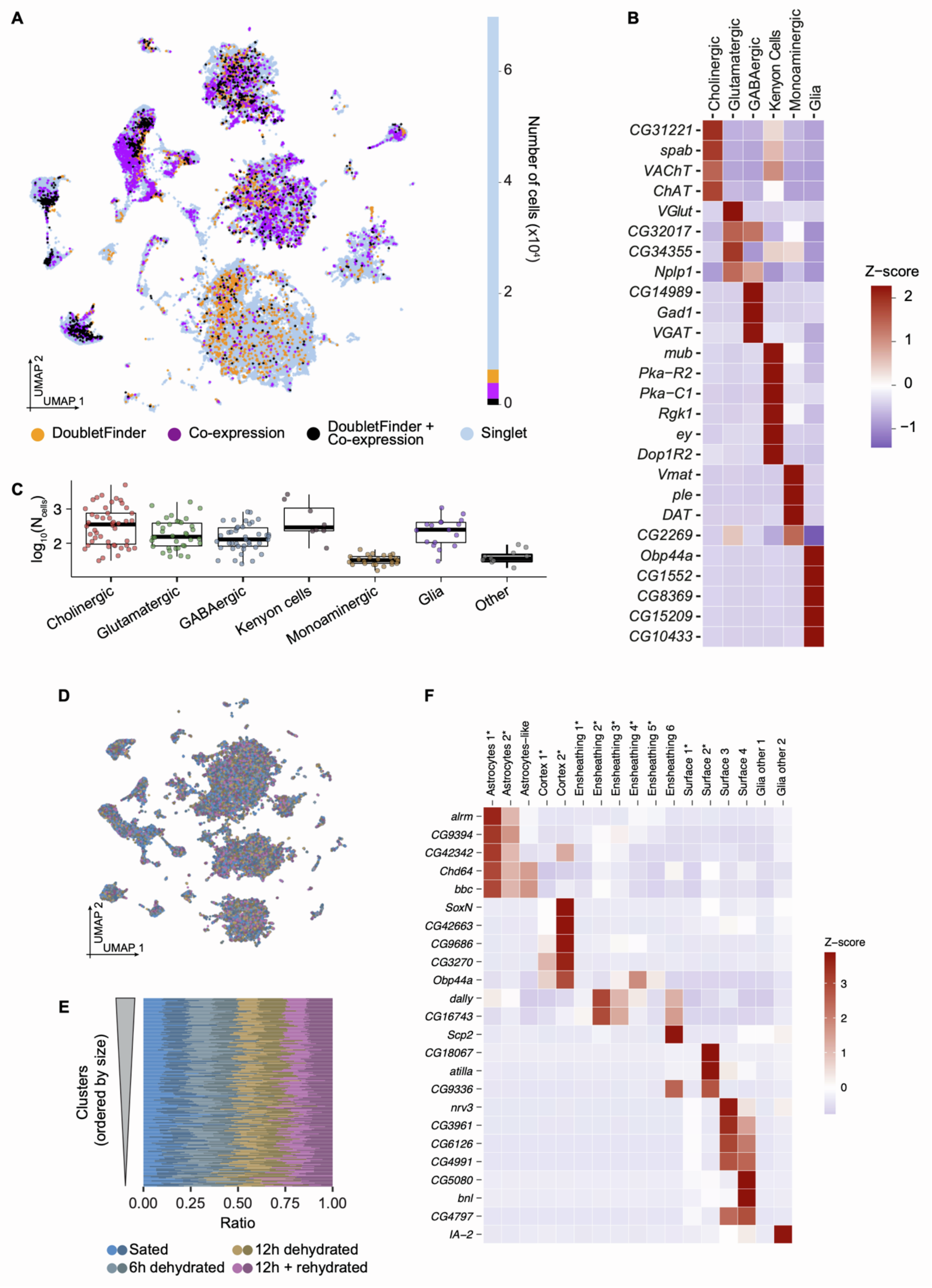

Figure S1. Filtering and clustering single-cell transcriptomics data, Related to Figure 1. (**A**) Left: UMAP plot obtained after initial clustering, showing doublets identified with DoubletFinder or the co-expression strategy. Right: barplot showing the number of cells

labelled with either method and that were discarded prior to subsequent analyses. (**B**) Heatmap showing expression of the top marker genes from six of the main cell classes. (**C**) Number of cells per cluster, grouped by main cell classes. (**D**) Same UMAP plot as Figure 1E, with colors representing the experimental sample from which each cell originates. (**E**) Ratio of cells originating from each sample, for each cluster, ordered by size. All clusters contain cells from each condition and sample. As expected, more variability was evident in the small clusters. (**F**) Heatmap showing expression of the top marker genes from each glia cluster.

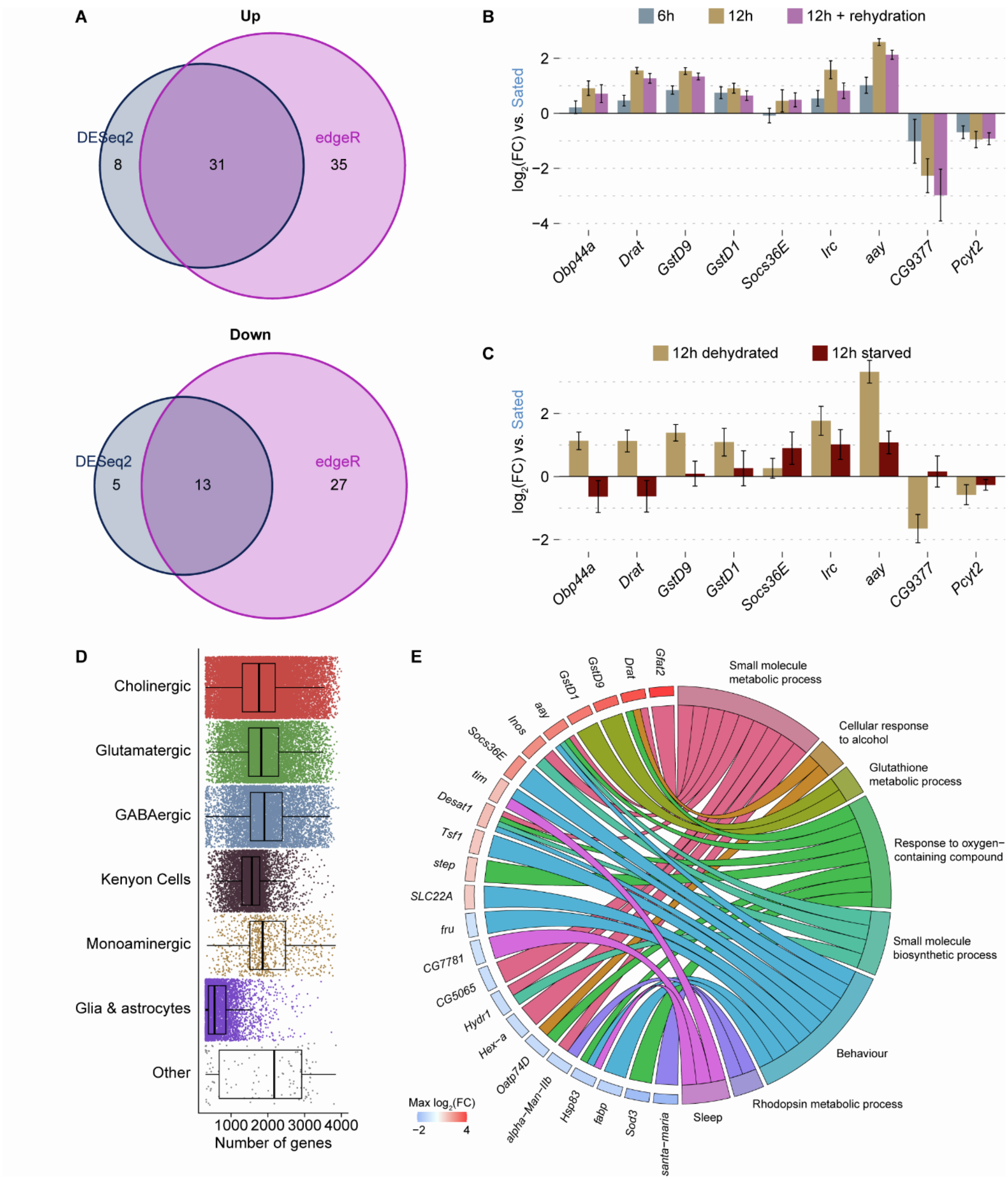

Figure S2. Differential gene expression in thirsty flies, Related to Figure 2.

(**A**) Venn diagrams showing the number of gene expression events identified with DESeq2, edgeR, or both methods. Top: up-regulated genes. Bottom: down-regulated genes. (**B**) Relative expression changes for a selection of differentially expressed genes in thirsty flies. Groups are those as in Figure 1B, and values are relative to those in sated flies. Data are from RT-qPCR of whole heads, n2-3. (**C**) Relative expression changes for a selection of genes that

were differentially expressed in thirsty flies, in a 12h dehydrated and 12 h starved condition, relative to levels in sated animals. RT-qPCR on whole heads, n = 2-3. (**D**) Number of genes detected in each cell, grouped by main class. (**E**) Chord diagram showing the most represented GO categories across the genes that are differently expressed in glia of thirsty flies.

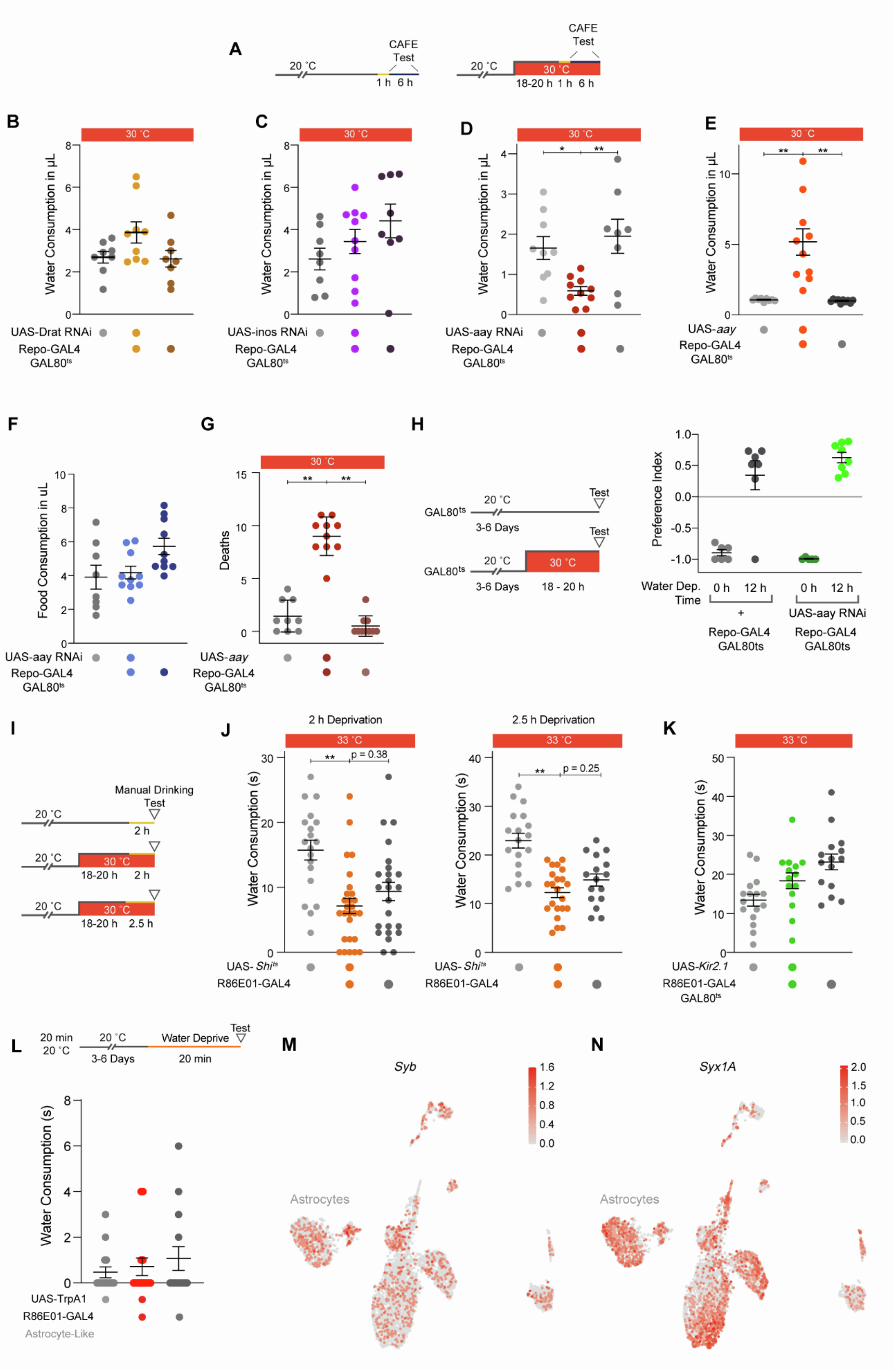

Figure S3. Extra water consumption and water preference data, Related to Figure 3.

(**A**) CAFE water consumption assay protocol. Orange section of black line indicates period when flies are water deprived. (**B**) Knockdown of *Drat* in glia does not change water consumption in CAFE. (**C**) Knockdown of *inos* in glia does not alter water consumption in CAFE. (**D**) Knockdown of *aay* in glia reduces water consumption in CAFE. (**E**) Overexpression of *aay* in glia increases water consumption in CAFE. (**F**) Knockdown of *aay* does not alter food consumption as measured in CAFE. (**G**) Number of deaths per vial of 12 flies from S1D during CAFE assay shows overexpression of *aay* makes flies more susceptible to water deprivation. (**H**) Flies with *aay*-knockdown in glia exhibit normal hygrosensation in the T-maze. Flies show normal innate avoidance of high humidity when water sated, and display normal water seeking when water deprived. (**I**) Temperature regimen for inhibiting astrocyte vesicle release with expression temperature sensitive *Shibire*^{ts} while measuring water consumption. (**J**) S*hibire*^{ts} expression in astrocytes does not significantly suppress water consumption, relative to controls. (**K**) Overexpressing the inward-rectifying potassium channel Kir2.1 in astrocytes did not significantly change water consumption. (**L**) Permissive temperature control for *R86E01>TrpA1* activation experiments (Figure show that flies that do not have activated astrocytes do not increase water consumption (n = 14, 15 flies). (**M**) UMAP plot showing expression levels of *Syb* across glial clusters. Scale represents normalized expression levels. Refer to Figure 1F for annotations. (**N**) UMAP plot showing expression levels of *Syx1A* across glial clusters. Scale represents normalized expression levels. Refer to Figure 1F for annotations. For panels B-H and J-K, normality was assessed using the Shapiro-Wilk test. * $p<0.05$, ** $p<0.01$, Ordinary one-way ANOVA with Dunnett's multiple comparisons test, unless otherwise stated. Data are mean +/- SEM.

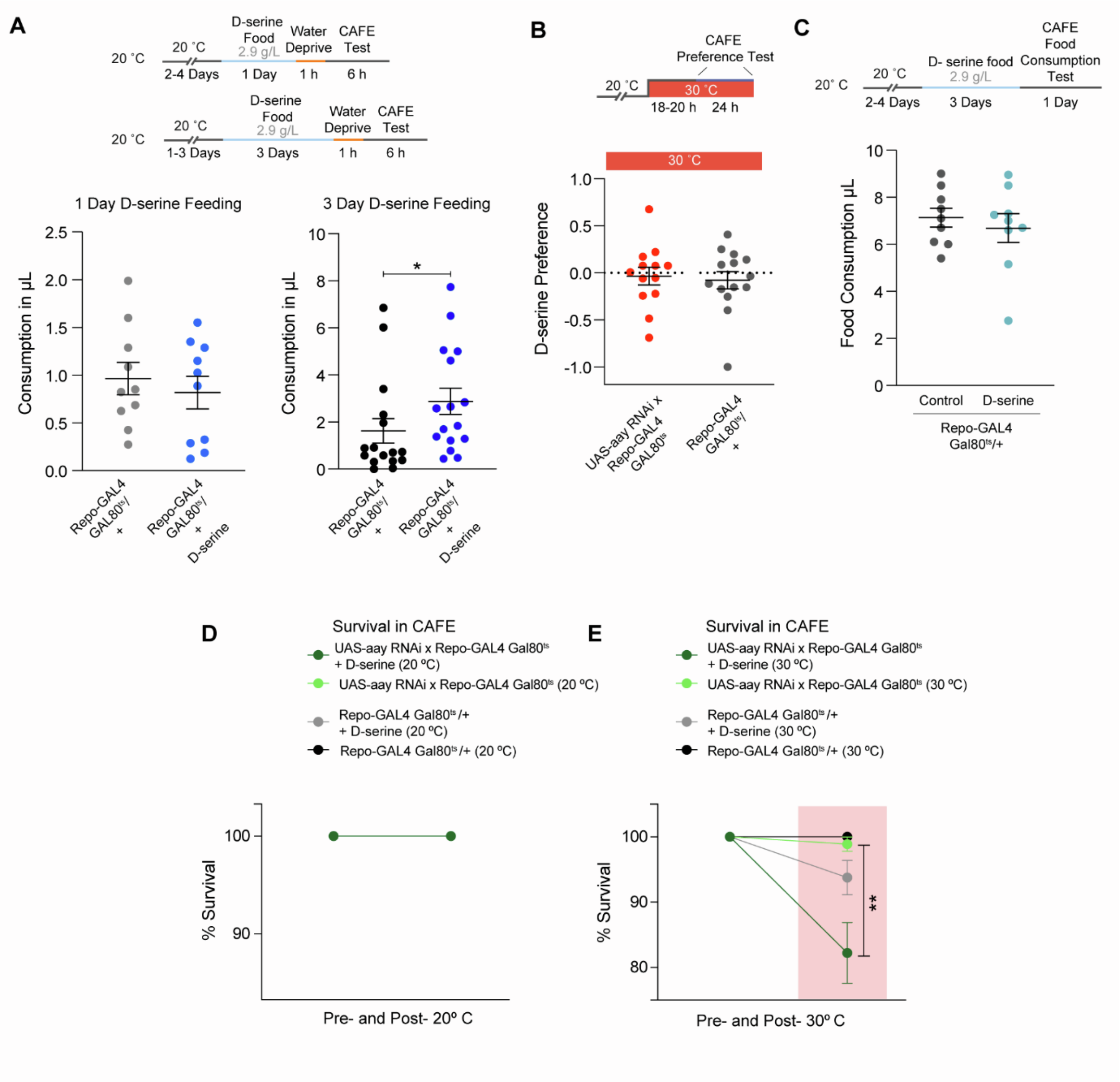

Figure S4. Effects of D-serine feeding and D-serine preference, Related to Figure 4.

(**A**) Protocol for D-serine feeing and testing in the CAFE assay and results from 1 and 3 days of D-serine feeding. Only 3 days of D-serine feeding significantly increased water consumption. * p<0.05 two-tailed Mann-Whitney test ($n_{1\text{-day}}$ = 10 vials, $n_{3\text{-days}}$ = 16 vials). (**B**) Neither *aay*-knockdown or GAL4/GAL80^{ts} control flies show a preference for D-serine containing food in CAFE (n = 13, 14 vials). (**C**) D-serine feeding does not change food consumption in the CAFE (n = 9 vials). (**D**) Survival of *aay*-knockdown flies in CAFE at permissive 20°C (no induction of *aay*-RNAi expression) that were either fed D-serine or kept on 'D-serine free' food. Flies survived throughout the assay (n = 8, 9 vials). (**E**) Survival of *aay*-knockdown flies in CAFE with a 30°C heat step (induced *aay*-RNAi expression). Flies with *aay* knockdown that were also fed D-serine show significantly compromised survival in CAFE (n = 8, 9 vials). ** p<0.01 *aay*-RNAi vs. *aay*-RNAi + D-serine. Normality was assessed using the

Shapiro-Wilk test. * p<0.05, ** p<0.01, *** p<0.001, **** p<0.0001 Ordinary one-way ANOVA with Dunnett's multiple comparisons test, unless otherwise stated. Data are mean +/- SEM.

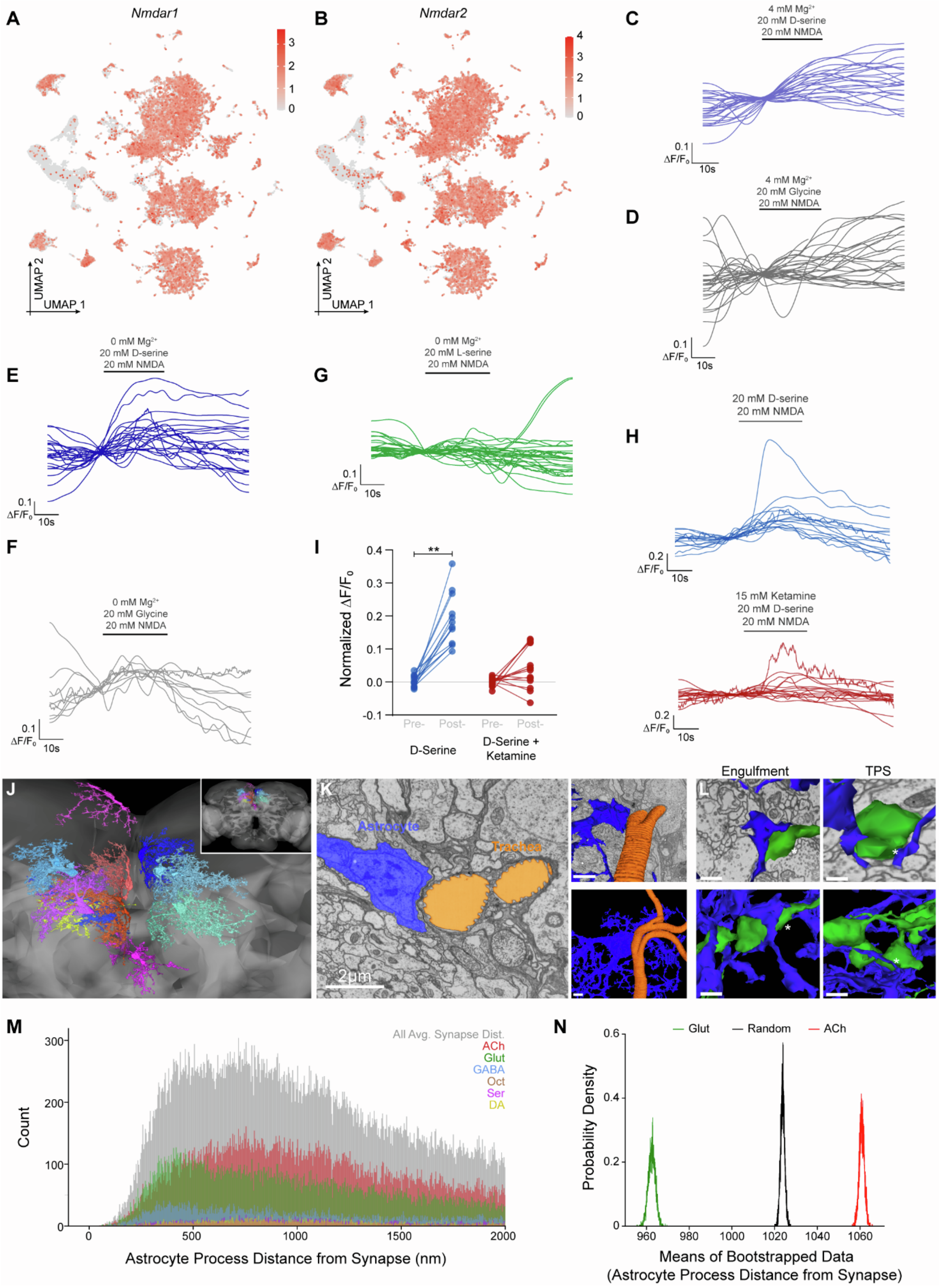

Figure S5. D-serine is a co-agonist for NMDARs, Related to Figure 5

(**A**) UMAP plot showing expression levels of *Nmdar1* across glial clusters. Scale represents normalized expression levels. Refer to Figure 1E for annotations. (**B**) UMAP plot showing

expression levels of *Nmdar2* across glia clusters. Scale represents normalized expression levels. Refer to Figure 1E for annotations. (**C**) Individual $\Delta F/F_0$ GCaMP7f traces of NR1-expressing neurons to D-serine and NMDA application in high Mg^{2+}. (**D**) Individual traces of NR1 neurons to glycine and NMDA application in high Mg^{2+}. (**E**) Individual traces of NR1 neurons to D-serine and NMDA application in 0 mM Mg^{2+}. (**F**) Individual traces of NR1 neurons to glycine and NMDA application in 0 mM Mg^{2+}. (**G**) Individual traces of NR1 neurons to L-serine and NMDA application in 0 mM Mg^{2+}. (**H**) Individual traces of NR1 neuron responses to D-serine and NMDA application, with and without ketamine. (**I**) quantification of Pre- and post-responses of Figure 5H with outliers removed. ** $p<0.01$, Kruskal-Wallis ANOVA with Dunn's multiple comparisons test. (**J**) 3D representation of 11 reviewed astrocytes with processes in the SMP. Brain neuropil is grey background. Astrocyte somata reside at the neuropil boundary and their processes are mostly mutually exclusive to one area of the neuropil. Few astrocyte processes venture past ensheathing glial boundaries. Inset shows lesser zoom for position of reference within the entire brain. (**K**) Astrocyte cell bodies are often close to trachea. Left: Greyscale EM data with a glial soma (blue) and two tracheal branches (orange). Upper right: Same greyscale image with 3D reconstructions of astrocyte cell body and trachea superimposed. Lower right: 3D renderings show the astrocyte in relation to trachea. (**L**) Same EM section as Figure 5I with superimposed 3D reconstruction of neuronal boutons and astrocytic processes engulfing a bouton (top left) and contributing to a tripartite synapse (TPS) (top right). Bottom left: 3D renderings show astrocytic processes engulf the synaptic bouton near the synapse (asterisk). Bottom right: 3D reconstructions show 3 boutons are contacted by thin astrocytic processes. Location of synapse from Fig 5I indicated (asterisk). Scale bars 300nm. See also Video S1. (**M**) Astrocytic processes are closer to glutamatergic synapses, than GABAergic and cholinergic synapses. This is shown in a vicinity profile for SMP astrocytes consisting of histograms of the distribution of distances between synapses and fine astrocytic processes (less than 300nm radius), sorted by predicted neurotransmitter type. Note: Engulfment happens typically within 500nm of synapses and astrocytess often also contact postsynaptic processes more distant from the synapse. (**N**) Astrocytes are significantly closer to glutamatergic than cholinergic synapses. Means of 10000 bootstrap samples taken from the probability distributions of distances between synapses and glial processes of the SMP astrocytes. Only glutamatergic and cholinergic synapses in the vicinity of fine astrocytic processes (2µm radius around processes with radius $\leq$ 300nm) are displayed together with random draws from both sets. All 3 distributions are significantly different from each other. Heteroscedastic t-test: $p = 0$ (Glut <> Random) and $p = 10^{-200}$ (ACh <> Random).

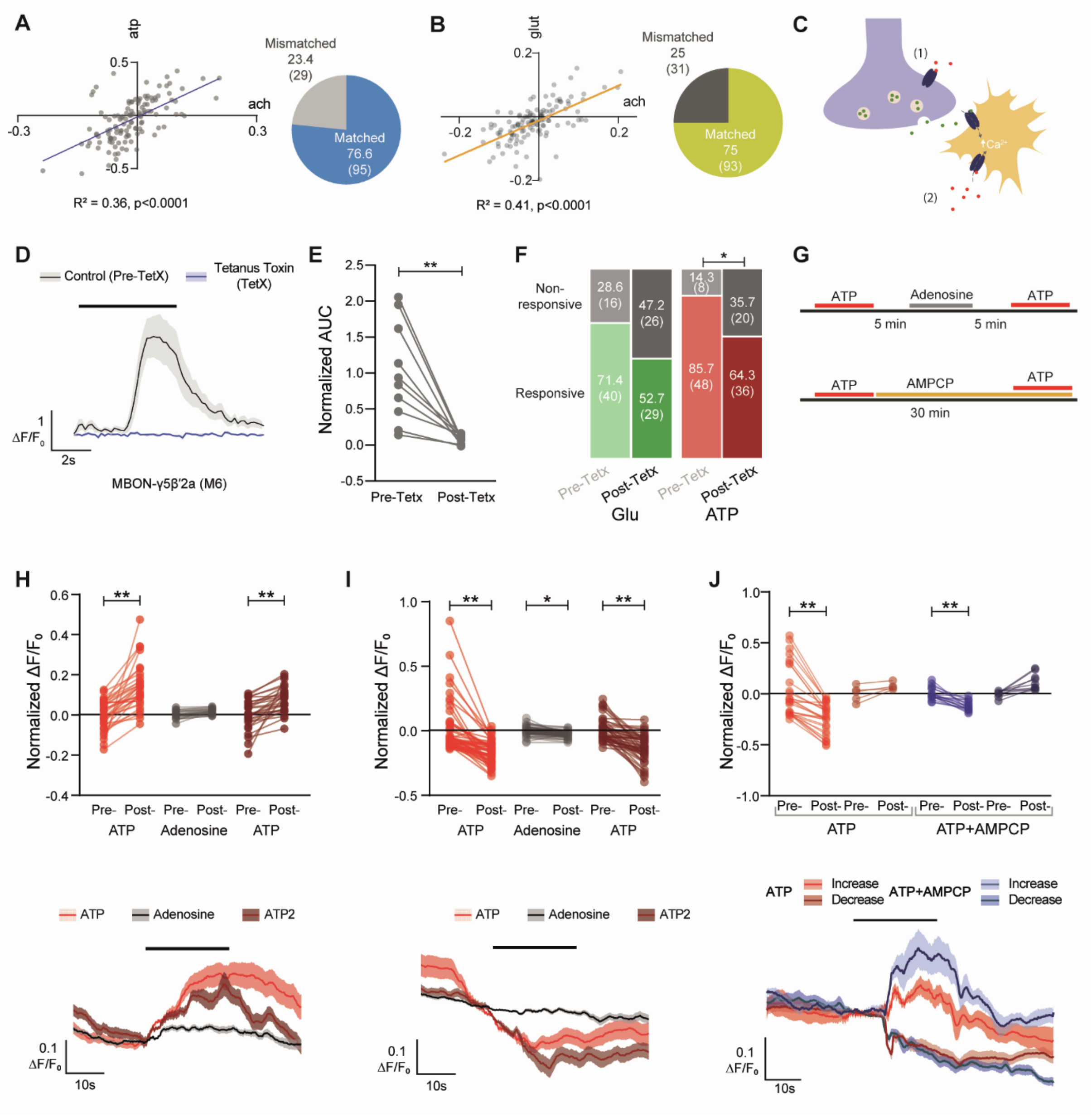

Figure S6. Specificity of astrocytic responses to neurotransmitters, Related to Figure 6

(**A**) Astrocytes show matched responses to ATP and ACh application. $\Delta F/F_0$ responses for ACh plotted against ATP responses with linear regression. Pie chart shows number of astrocytes with matched and mismatched responses between ACh and ATP. Fisher's exact test matched vs. mismatched, p = 1.076 $*10^{-5}$ OR = 3.28. (**B**) Astrocytes show matched responses to glutamate and ACh application. $\Delta F/F_0$ responses for glutamate plotted against ACh responses with linear regression. Pie chart shows number of astrocytes with matched and mismatched responses between glutamate and ACh. Fisher's exact test matched vs. mismatched, p = 2.27 $*10^{-6}$ OR = 3.72. (**C**) Schematic of two possible mechanisms for differential astrocytic responses with experimental procedure. 1) Exogenously applied neurotransmitter (red dots) acts on pre-synaptic or any non-astrocytic compartment that drives

release of a secondary neurotransmitter (green dots, which then drives changes in astrocytic Ca^{2+}. 2) Exogenously applied neurotransmitter (red dots) acts directly on the astrocytes modulating Ca^{2+} levels. Co-applying tetanus toxin with exogenous neurotransmitter application should prevent mechanism 1) from occurring. (**D**) 1 h of 1μM Tetanus toxin (TetX) application is sufficient to suppress polysynaptic activity. Odor exposure (black bar) to the fly activates MBON-γ5β′2a (M6). Tetx suppresses odor-induced activity recorded in the β′2a region of the MBON-γ5β′2a dendrite. (**E**) Area Under the Curve (AUC) analysis of β′2a dendritic field before and after Tetx application shows elimination of odor-induced Ca^{2+} response. ** $p<0.01$ paired t-test. (**F**) Most glutamate and ATP responsive astrocytes retain their neurotransmitter sensitivity following Tetx application. Tetx application reduced the number of ATP sensitive astrocytes and modestly reduced glutamate responsive astrocytes ($p = 0.066$). * $p<0.05$ Fisher's exact test with Bonferroni correction. (**G**) Protocol for ATP and adenosine application, and ATP with AMPCP. (**H**) Astrocytes show excitatory responses to ATP but not to adenosine. ** $p<0.01$, Kruskal-Wallis ANOVA with Dunn's multiple comparisons test. (**I**) Astrocytes show inhibitory responses to ATP and adenosine. * $p<0.05$, ** $p<0.01$, Kruskal-Wallis ANOVA with Dunn's multiple comparisons test. (**J**) Inhibiting ATP degradation by blocking ecto-5′-nucleotidase/CD73 with AMPCP does not abrogate ATP responses ** $p<0.01$, Kruskal-Wallis ANOVA with Dunn's multiple comparisons test.

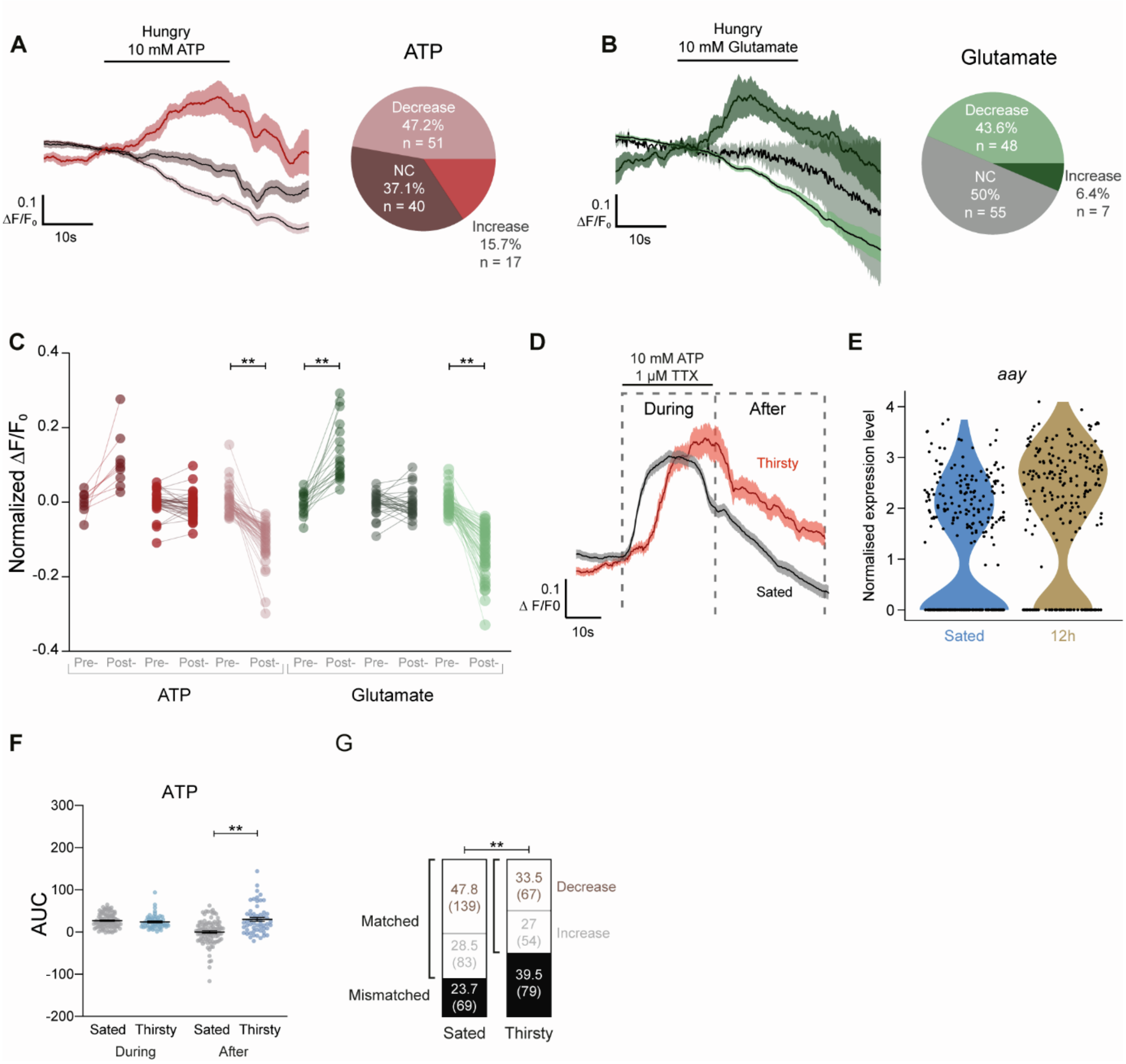

Figure S7. Astrocytic responses to ATP and glutamate in hungry and thirsty flies, Related to Figure 7

(**A and B**) Astrocytes show differential responses to ATP and glutamate in hungry flies. Bold lines are mean responses and shade is SEM. Pie charts show proportion of astrocytes that show positive, no change (NC), and negative responses to ATP and glutamate. (**C**) Quantification of $\Delta F/F_0$ before and after neurotransmitter application in hungry flies. ** p<0.01, Kruskal-Wallis ANOVA with Dunn's multiple comparisons test. (**D**) Ca^{2+} imaging traces of positive responsive astrocytes in thirsty and sated animals with ATP application. Line is mean and fill SEM. (**E**) Violin plot showing the difference of *aay* expression in all astrocytes in sated and 12h dehydrated flies. Each dot represents an individual cell. (F) Area Under the Curve (AUC) of excitatory ATP responses of astrocytes in sated vs. thirsty animals. ** p<0.01, Kruskal-Wallis ANOVA with Dunn's multiple comparisons test.

(G) Water deprivation desynchronizes matched astrocyte responses for glutamate and ATP. ** $p<0.01$, Fisher's exact test with Bonferroni correction.

Video S1. Fine astrocytic processes often engulf glutamatergic synaptic boutons or contribute to tripartite synapses, Related to Figures 5I and S5L. 3D representations of an astrocyte (blue) in the SMP and glutamatergic neurons (green) with boutons containing annotated presynapses (red), which consist of synaptic vesicles (red spheres) and pre-synaptic T-bar densities (black). 00:00 to 00:55, When glial cells engulf axons, multiple processes of different sizes contact a substantial area of a presynaptic bouton's membrane but do not extend into the synapse. 00:56-1:40, Fine astrocyte processes either pass through the synaptic cleft, or terminate within it, to form a tripartite synapse. In both cases the astrocyte processes contact the synaptic cleft alongside the postsynaptic neurons. Many closely spaced boutons of the same presynaptic neuron can be contacted by the same astrocyte, which therefore contributes processes to multiple tripartite synapses. Anatomical features are further highlighted in the video. Cellular meshes were retrieved from FlyWire and animations produced with blender

Data S1. Top marker genes for each cluster in the entire data set, Related to Figure 1 and Figure S1.

Data S2. Top markers for glial clusters, Related to Figure 1 and Figure 2.
